# Supplementary material for: Prosaposin maintains lipid homeostasis in dopamine neurons and counteracts experimental parkinsonism in rodents
Source: Nat Commun. 2023 Sep 19;14:5804. doi: 10.1038/s41467-023-41539-5 (PMC10509278; doi:10.1038/s41467-023-41539-5)
Supplement: Supplementary file 1 — Supplementary Information [file 41467_2023_41539_MOESM1_ESM.pdf]

SUPPLEMENTARY INFORMATION

Prosaposin maintains lipid homeostasis in dopamine neurons and counteracts experimental Parkinsonism in rodents

This file contains:

1. Supplementary Figures and Legends
2. Supplementary Tables
3. Supplementary Discussion
4. Supplementary References

1. Supplementary Figures and Legends

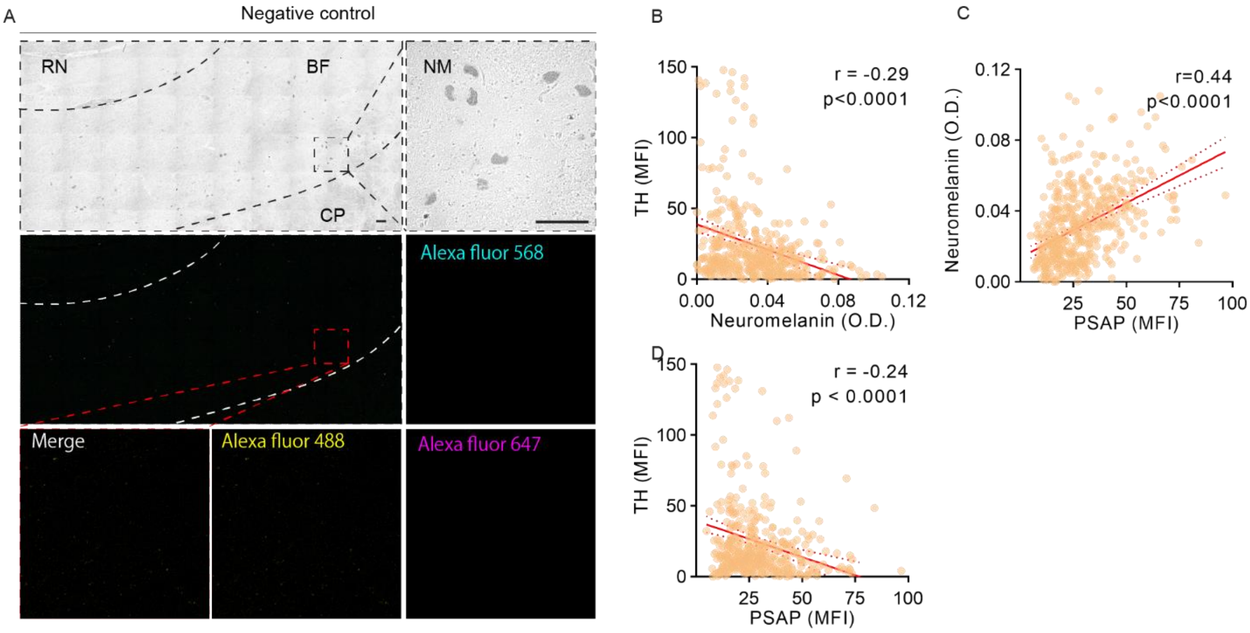

Supplementary Fig. 1. Negative control of immunofluorescent staining on postmortem human substantia nigra section and association of PSAP with neuromelanin. (**A**) Representative images of negative control immunofluorescent staining on postmortem human substantia nigra sections only with donkey anti-mouse IgG Alexa Fluor 488, donkey anti-goat IgG Alexa Fluor 568, and donkey anti-rabbit IgG Alexa Fluor 647 secondary antibodies. No primary antibodies were applied. BF, bright field; RN, red nucleus; CP, cerebral peduncle; NM, neuromelanin. Scale bars, 100 μm. (**B-D**) Scatter plot representing the associations of neuromelanin with TH (B), PSAP with neuromelanin (C), and PSAP with TH (D) levels in TH or neuromelanin positive neurons on postmortem human substantia nigra sections. The correlation coefficients (r) and the *p*-values are calculated by Pearson correlation. The solid and dashed lines indicate the linear regression line and the 95% confidential interval (CI), respectively. Each dot represents one neuron. N=430.

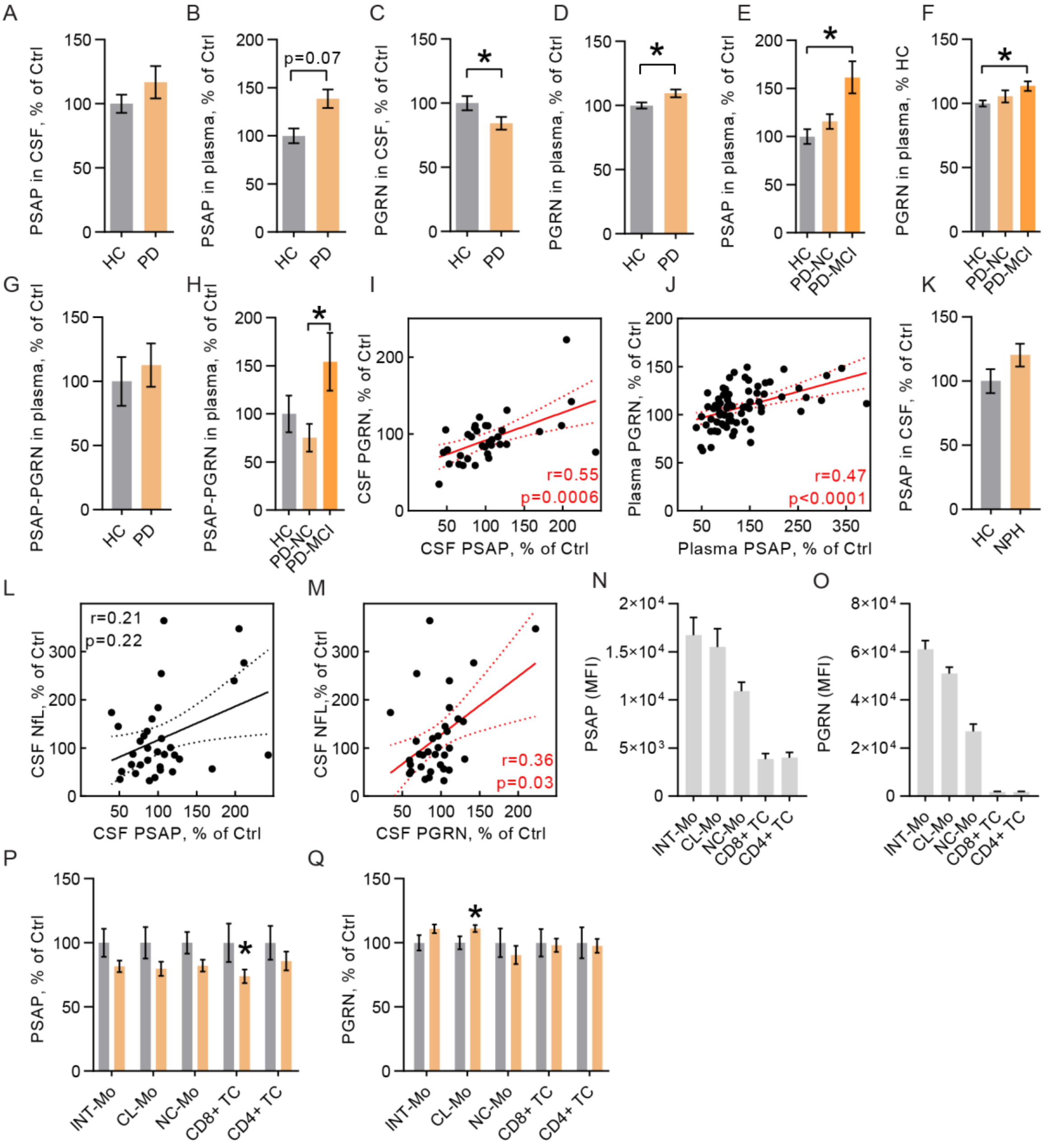
A
PSAP in CSF, % of Ctrl
HC
PD
B
PSAP in plasma, % of Ctrl
p=0.07
HC
PD
C
PGRN in CSF, % of Ctrl
*
HC
PD
D
PGRN in plasma, % of Ctrl
*
HC
PD
E
PSAP in plasma, % of Ctrl
*
HC
PD-NC
PD-MCI
F
PGRN in plasma, % HC
*
HC
PD-NC
PD-MCI
G
PSAP-PGRN in plasma, % of Ctrl
HC
PD
H
PSAP-PGRN in plasma, % of Ctrl
*
HC
PD-NC
PD-MCI
I
CSF PGRN, % of Ctrl
CSF PSAP, % of Ctrl
r=0.55
p=0.0006
J
Plasma PGRN, % of Ctrl
Plasma PSAP, % of Ctrl
r=0.47
p<0.0001
K
PSAP in CSF, % of Ctrl
HC
NPH
L
CSF NfL, % of Ctrl
CSF PSAP, % of Ctrl
r=0.21
p=0.22
M
CSF NFL, % of Ctrl
CSF PGRN, % of Ctrl
r=0.36
p=0.03
N
PSAP (MFI)
INT-Mo
CL-Mo
NC-Mo
CD8+ TC
CD4+ TC
O
PGRN (MFI)
INT-Mo
CL-Mo
NC-Mo
CD8+ TC
CD4+ TC
P
PSAP, % of Ctrl
*
INT-Mo
CL-Mo
NC-Mo
CD8+ TC
CD4+ TC
Q
PGRN, % of Ctrl
*
INT-Mo
CL-Mo
NC-Mo
CD8+ TC
CD4+ TC

Supplementary Fig. 2. Changes of PSAP and PGRN levels in CSF, plasma, and PBMCs of PD patients. (**A-B**) PSAP levels in CSF (A) and plasma (B) from healthy controls (HCs) and PD patients. N=15, 21 in (A), N=17, 60 in (B). (**C-D**) PGRN levels in CSF (C) and plasma (D) of HCs and PD patients. N=16, 21 in (C), N=19, 60 in (D). (**E**) PSAP levels in plasma from HCs, PD with normal cognition (PD-NC), and PD with mild cognitive impairment (PD-MCI). N=17, 30, 30. (**F**) PGRN levels in the plasma of HCs, PD-NCs, and PD-MCIs. N=19, 30, 30. (**G**) PSAP-PGRN complex levels in plasma from HCs and PD patients. N=19, 59. (**H**) PSAP-PGRN complex levels in plasma from HCs, PD-NCs, and PD-MCIs. N=19, 31, 28. (**I-J**) Scatter plots representing the associations of PSAP with PGRN in CSF (I) and plasma (J). Each dot depicts a CSF or plasma PSAP value and their corresponding PGRN value of one HC or PD patient. N=35, 74 for (I) and (J), respectively. (**K**) PSAP levels in CSF of HCs and NPH patients. N=12, 11. (**L-M**) Scatter plots representing the associations of CSF PSAP (L) or PGRN (M) with CSF NfL. Each dot depicts a CSF PSAP (L) or PGRN (M) value and their corresponding NfL value of one HC or PD patient. N=33, 34 for (L) and (M), respectively. (**N-O**) PSAP (N) and PGRN (O) levels in intermediate monocytes (INT-Mo), classical monocytes (CL-Mo), non-classical monocytes (NC-Mo), $CD8^+$ T cells ($CD8^+$ TC), and $CD4^+$ T cells ($CD4^+$ TC), measured as mean fluorescence intensity (MFI). N=15 controls in (N), N=14 controls in (O). (**P-Q**) PSAP (P) and PGRN (Q) levels in INT-Mo, CL-Mo, NC-Mo, $CD8^+$ TC, and $CD4^+$ TC of HC and PD patients. N=15 controls, 31 PD patients in (P), N=14 controls, 36 PD patients in (Q). Data are normalized to the mean of the control group as indicated and presented as mean ± S.E.M. Mann-Whitney test (A-B, D-H), Student's t-test (C, K, P-Q). Pearson correlation coefficients (r) and *p*-values are calculated in (J), and nonparametric Spearman correlation r and *p*-values were calculated in (I, L, M). The solid and dashed lines indicate the simple linear regression line and the 95% confidential interval (CI), respectively. Non-significant *p* value is not labelled, $*p<0.05$.

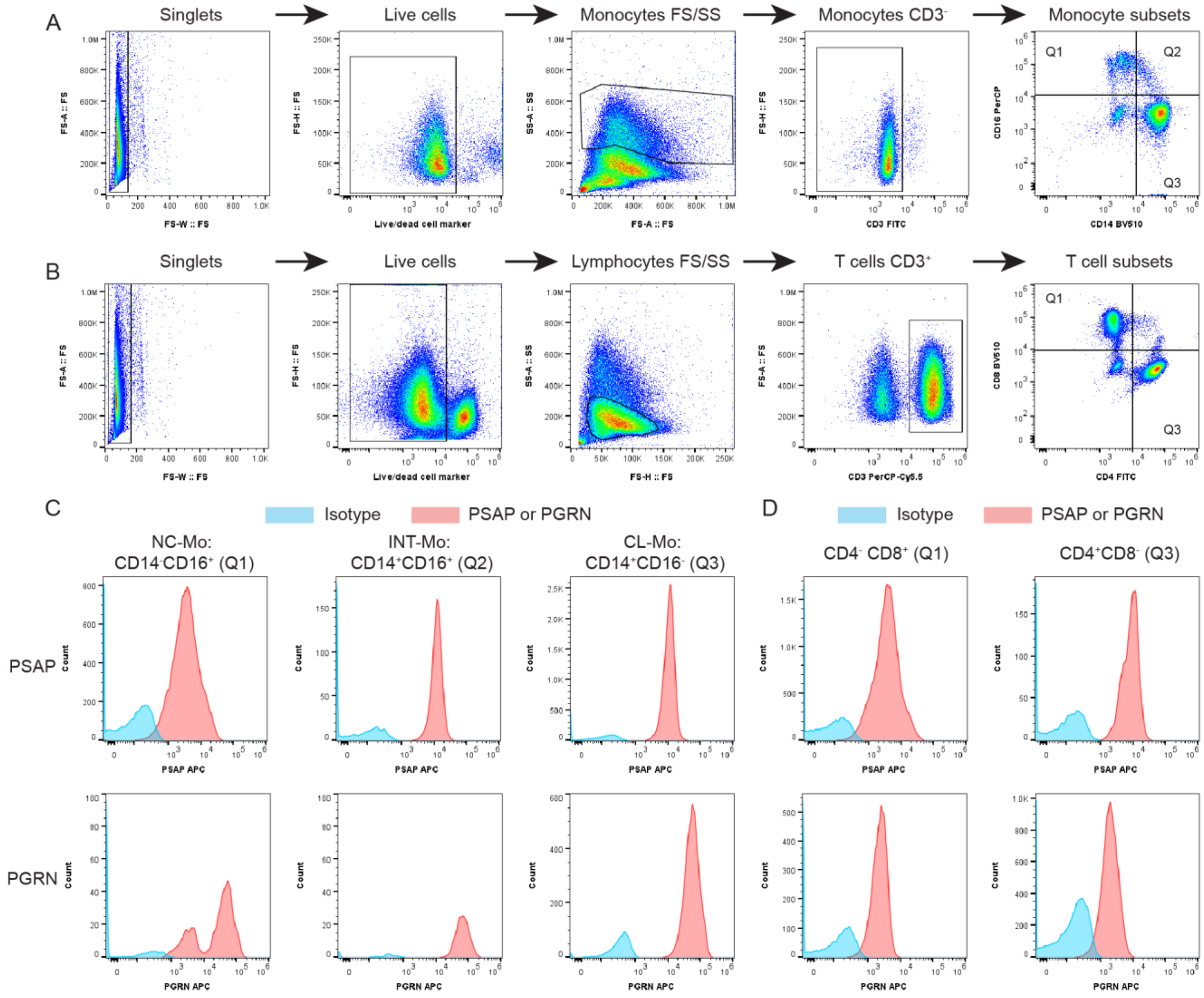
A
Singlets
Live cells
Monocytes FS/SS
Monocytes CD3⁻
Monocyte subsets
FS-A :: FS
FS-W :: FS
FS-H :: FS
Live/dead cell marker
SS-A :: SS
FS-A :: FS
CD3 FITC
CD16 PerCP
CD14 BV510
Q1
Q2
Q3
B
Singlets
Live cells
Lymphocytes FS/SS
T cells CD3⁺
T cell subsets
FS-W :: FS
Live/dead cell marker
FS-H :: FS
CD3 PerCP-Cy5.5
CD8 BV510
CD4 FITC
Q1
Q3
C
Isotype
PSAP or PGRN
NC-Mo: CD14⁻CD16⁺ (Q1)
INT-Mo: CD14⁺CD16⁺ (Q2)
CL-Mo: CD14⁺CD16⁻ (Q3)
PSAP
PGRN
Count
PSAP APC
PGRN APC
D
Isotype
PSAP or PGRN
CD4⁻ CD8⁺ (Q1)
CD4⁺CD8⁻ (Q3)

Supplementary Fig. 3. Gating strategies of monocytes and T cells, and PSAP and PGRN antibody specificity in flow cytometry analyses. (A) Representative flow cytometry monocytes gating strategy plots. Black arrows indicate the order of the gating. The positive gating included singlets; exclusion of dead cells; approximate monocytes gating using size and granularity parameters; exclusion of $CD3^+$ cells; selected monocytes were finally gated into $CD14^-CD16^+$ (Q1, NC-Mo), $CD14^+CD16^+$ (Q2, INT-Mo) and $CD14^+CD16^-$ (Q3, CL-Mo) subsets. (**B**) Representative flow cytometry T cells gating strategy plots. Black arrows indicate the order of the gating. The positive gating included singlets; exclusion of dead cells; approximate lymphocyte gating using size and granularity parameters; exclusion of $CD3^-$ cells; selected lymphocytes were finally gated into $CD3^+CD8^+$ (Q1, $CD8^+$ TC) and $CD3^+CD4^+$ (Q3, $CD4^+$ TC) subsets. (**C**) Monocyte subsets (INT-Mo, CL-Mo, NC-Mo) intracellular staining of PSAP (top panel) or PGRN (bottom panel), and isotype control are illustrated with red and blue curves, respectively. (**D**) T cell subsets ($CD8^+$ TC, $CD4^+$ TC) intracellular staining of PSAP (top panel) or PGRN (bottom panel) and isotype control are illustrated with red and blue curves, respectively.

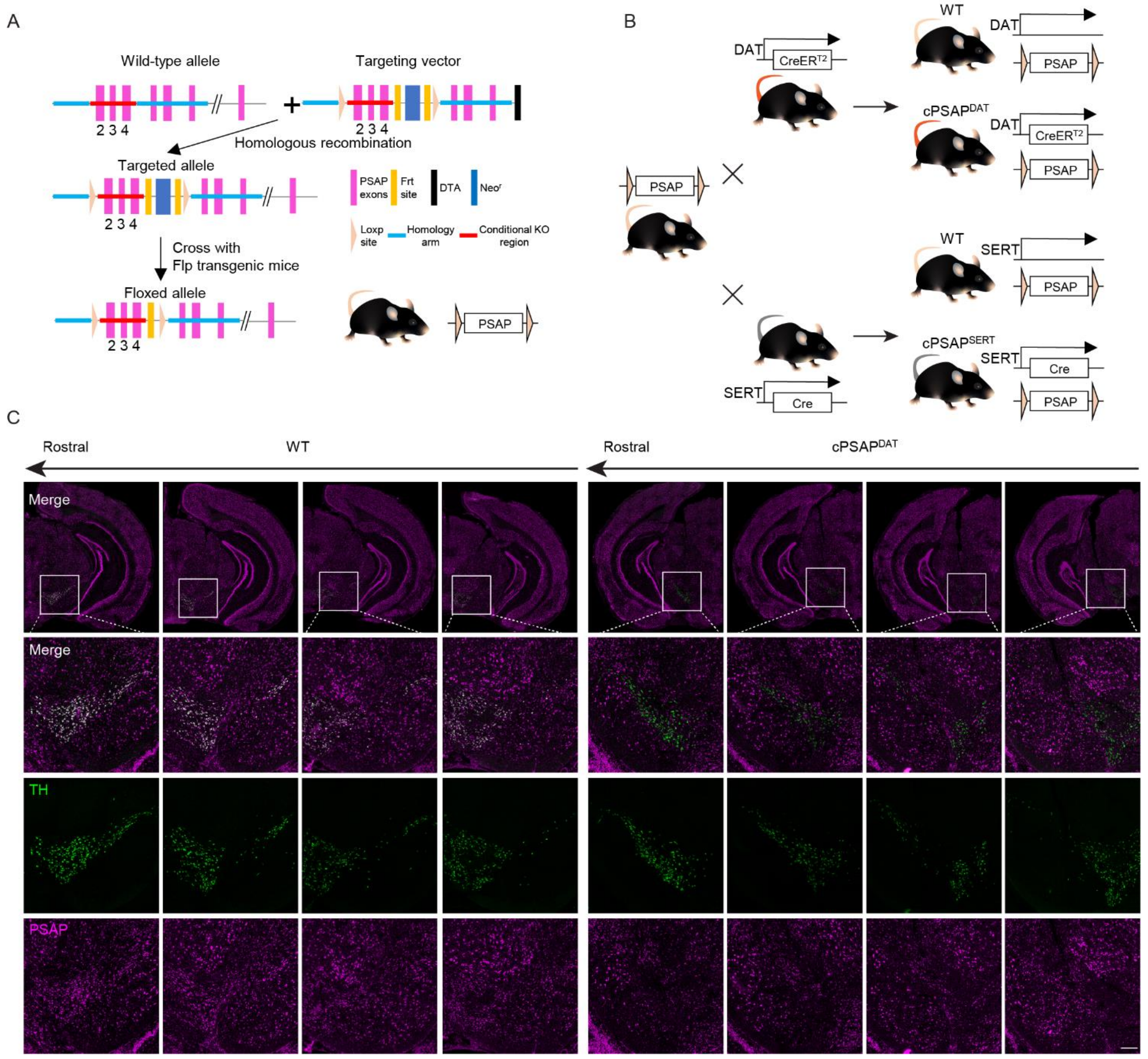
A
Wild-type allele
Targeting vector
2 3 4
2 3 4
Homologous recombination
Targeted allele
2 3 4
Cross with
Flp transgenic mice
Floxed allele
2 3 4
PSAP exons
Frt site
DTA
Neo
Loxp site
Homology arm
Conditional KO region
PSAP
B
WT
DAT
CreERT2
PSAP
cPSAPDAT
WT
SERT
cPSAPSERT
Cre
C
Rostral
WT
cPSAPDAT
Merge
TH
PSAP

Supplementary Fig. 4. Generation of floxed-PSAP mice and subsequently cPSAP^{DAT} and cPSAP^{SERT} mice

(**A**) Generation of floxed-PSAP mice. Using embryonic stem (ES) cell targeting technique, homologous recombination occurs between wild-type PSAP allele and targeting vector carrying homology arms and floxed exons (2-4) of PSAP. ES cell clones with targeted alleles are selected to generate mice with targeted alleles. By crossing these mice with Flp transgenic mice, mice with floxed PSAP are generated. (**B**) Generation of cPSAP^{DAT}, cPSAP^{SERT} mice, and corresponding WT control mice. PSAP-floxed mice are crossed with DAT-creER^{T2} mice and SERT-Cre mice, respectively. (**C**) Representative fluorescence in situ hybridization (FISH) images of TH mRNA (green) and PSAP mRNA (magenta) in the whole substantia nigra (12 µm thick sections, every 14th section) of WT and cPSAP^{DAT} mice. Scale bar, 200µm.

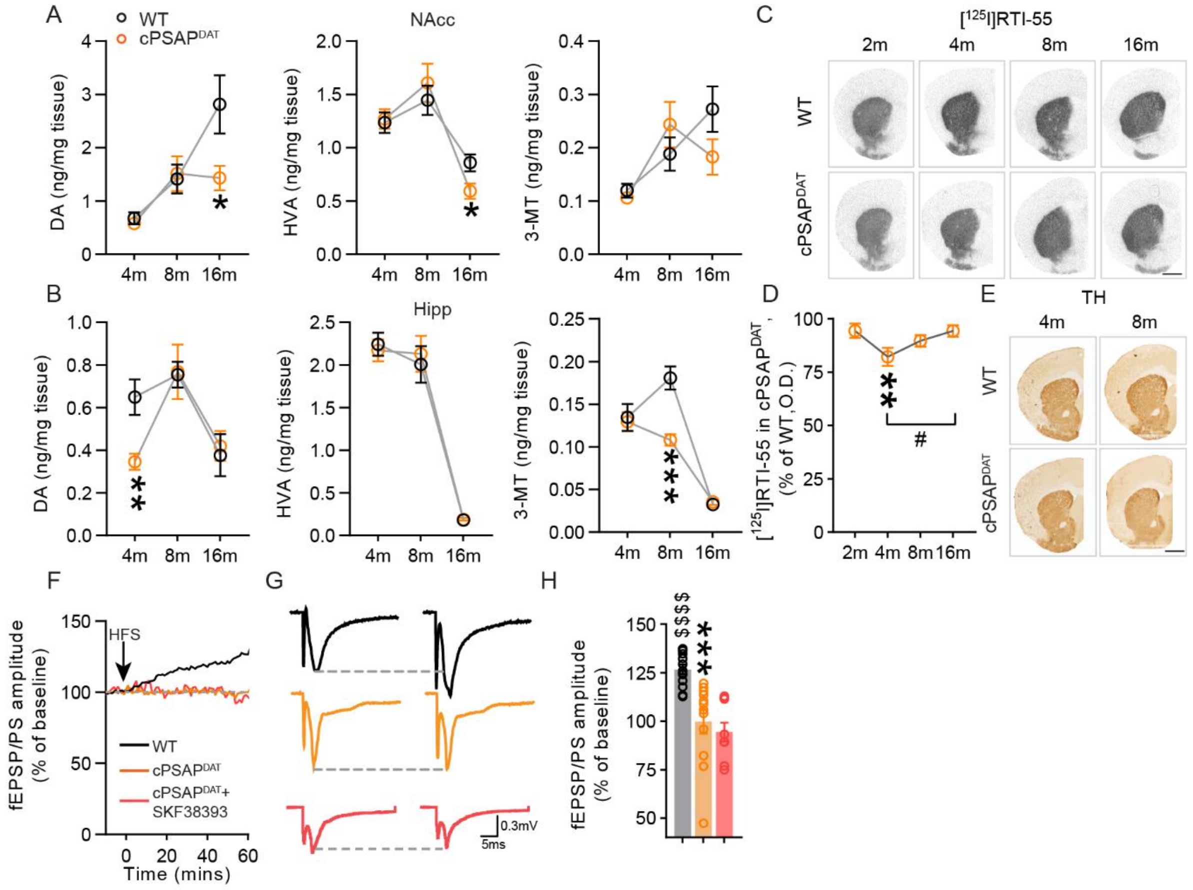
A
WT
cPSAP^DAT
NAcc
DA (ng/mg tissue)
HVA (ng/mg tissue)
3-MT (ng/mg tissue)
4m 8m 16m
C
[125I]RTI-55
2m 4m 8m 16m
WT
cPSAP^DAT
B
Hipp
D
[125I]RTI-55 in cPSAP^DAT (% of WT,O.D.)
2m 4m 8m 16m
#
E
TH
4m 8m
F
HFS
fEPSP/PS amplitude (% of baseline)
Time (mins)
WT
cPSAP^DAT
cPSAP^DAT+ SKF38393
G
0.3mV
5ms
H
$$$$
fEPSP/PS amplitude (% of baseline)

Supplementary Fig. 5. Neurochemical characterization and impaired striatal synaptic plasticity of cPSAP^{DAT} mice. (**A-B**) Line charts showing HPLC measurements of DA, HVA, and 3-MT in the nucleus accumbens (NAcc) (A) and hippocampus (Hipp) (B) of WT and cPSAP^{DAT} mice of 4m-, 8m- and 16m-old. N_{WT} =7, 7, 8-9, $N_{cPSAP^{DAT}}$ =8-10, 9-10, 13-16, respectively for three time points. (**C-D**) Representative autoradiographs (C) and quantification (D) of [^{125}I] RTI-55 binding with DAT on striatal sections of WT and cPSAP^{DAT} mice of 2m-, 4m-, 8m- and 16m-old. N_{WT} =7, 7, 7, 8 and $N_{cPSAP^{DAT}}$ =6, 10, 10, 16, respectively for four time-points. (**E**) Representative images of TH immunohistochemical staining in striatal sections of 4m- and 8m-old WT and cPSAP^{DAT} mice. (**F**) Time-course of the fEPSP/PS amplitude recorded in striatal slices of 4m-old WT and cPSAP^{DAT} (with or without SKF38393) mice. High-frequency stimulation (HFS) was applied at the time indicated by the arrow. N=13 slices from WT, 12 and 11 slices from cPSAP^{DAT} mice without and with SKF38393 treatment respectively. (**G**) Representative fEPSP/PS traces in WT (gray) and cPSAP^{DAT} (orange) mice before and after the HFS. (**H**) Dot plot presenting the average fEPSP/PS amplitude in slices from WT, cPSAP^{DAT} mice, and cPSAP^{DAT} mice treated with SKF38393, 55-60 min after HFS. Each circle represents one slice. N=13 slices from WT, 12 and 9 slices from cPSAP^{DAT} mice without and with SKF38393 treatment respectively. Scale bars, 1mm. Data are presented as mean ± S.E.M. Data in (D) is normalized to the means of WT controls. Student's t-test (A, B, H), or two-way ANOVA with Bonferroni's *post hoc* test (D) was applied appropriately. * Compared to WT, $^{\$}$ compared to WT baseline, $^{\#}$ compared to another time-point; $^{*/\#}p<0.05$, $^{**}p<0.01$, $^{***}p<0.001$, $^{\$\$\$\$}p<0.0001$.

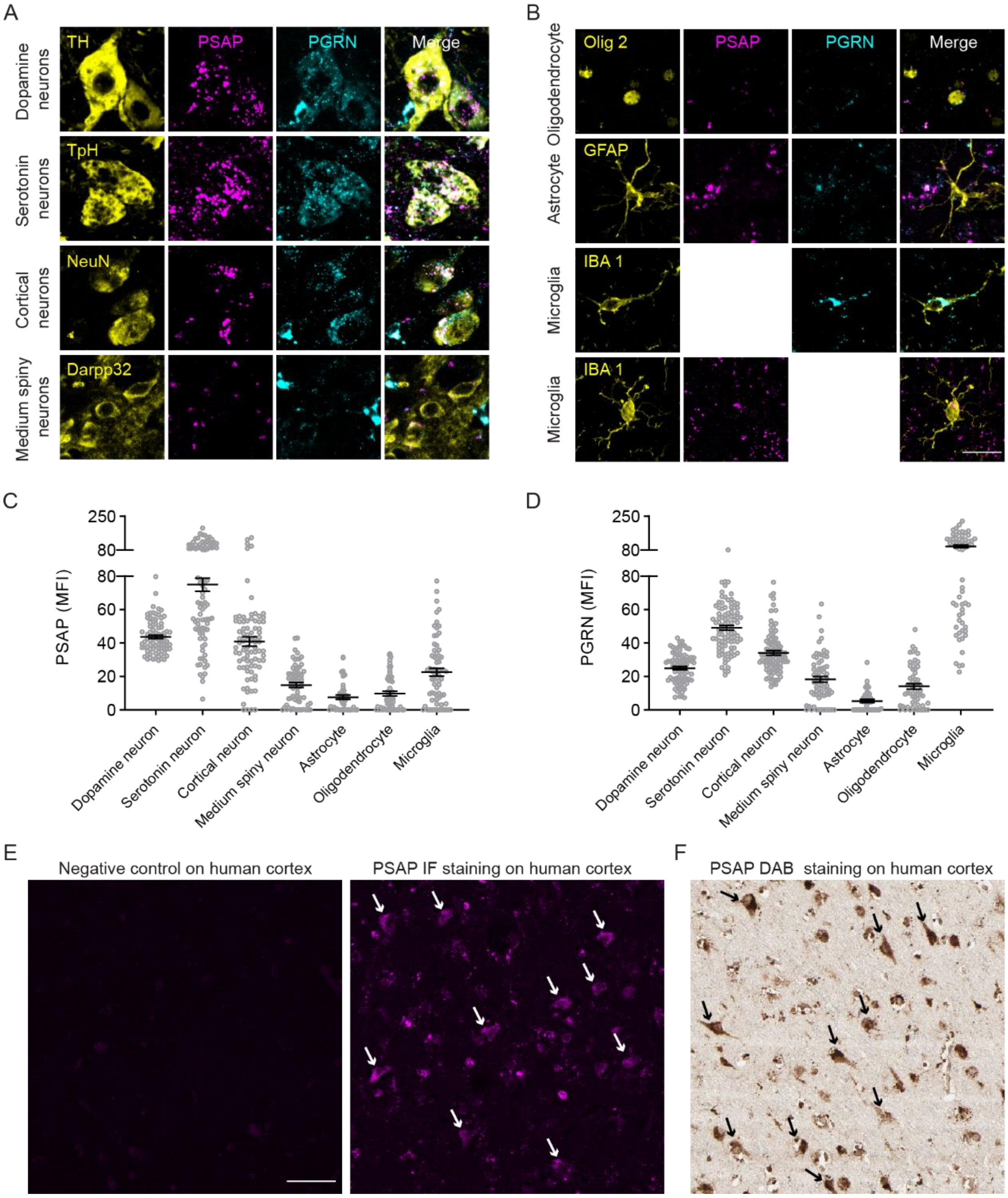

A
TH
PSAP
PGRN
Merge
Dopamine neurons
TpH
Serotonin neurons
NeuN
Cortical neurons
Darpp32
Medium spiny neurons
B
Olig 2
PSAP
PGRN
Merge
Oligodendrocyte
GFAP
Astrocyte
IBA 1
Microglia
IBA 1
Microglia
C
PSAP (MFI)
250
80
80
60
40
20
0
Dopamine neuron
Serotonin neuron
Cortical neuron
Medium spiny neuron
Astrocyte
Oligodendrocyte
Microglia
D
PGRN (MFI)
250
80
80
60
40
20
0
Dopamine neuron
Serotonin neuron
Cortical neuron
Medium spiny neuron
Astrocyte
Oligodendrocyte
Microglia
E
Negative control on human cortex
PSAP IF staining on human cortex
F
PSAP DAB staining on human cortex

Supplementary Fig. 6 Expression of PSAP and PGRN in different cell types of the mouse brain and neuronal PSAP expression in the human cortex (**A-D**) Representative images (A-B) and mean fluorescent intensity (MFI) quantification (C-D) of PSAP and PGRN immunofluorescent staining in dopamine (TH), serotonin (TpH), cortical (NeuN), medium spiny neurons (Darpp32), and oligodendrocytes (Olig2), astrocytes (GFAP), and microglia (IBA1). Scale bar, 20 µm. In (C-D), each circle represents one cell. Data are presented as mean ± S.E.M and N=81, 92, 83, 65, 44, 55, 68 cells in seven groups, respectively. © Representative PSAP immunofluorescent staining and negative control on human cortex section; white arrows indicate neuronal PSAP according to morphology; scale bar, 50 µm. (**F**) Image obtained from proteinatlas.org showing neuronal PSAP in human cortex with DAB staining; black arrows indicate neuronal PSAP according to morphology.

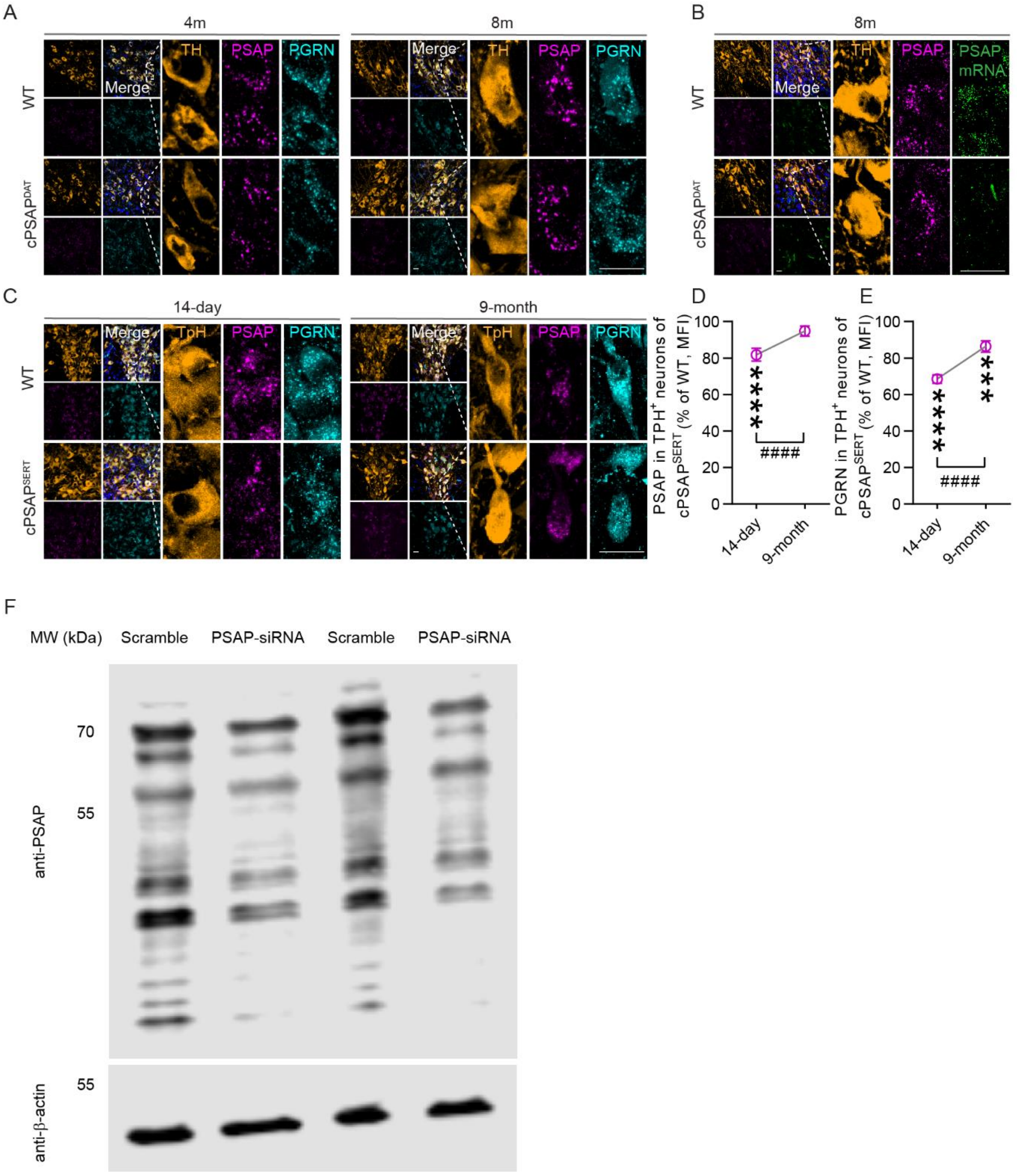
A
4m
8m
WT
cPSAP^DAT
Merge
TH
PSAP
PGRN
B
8m
PSAP mRNA
C
14-day
9-month
cPSAP^SERT
TpH
D
PSAP in TPH+ neurons of cPSAP^SERT (% of WT, MFI)
E
PGRN in TPH+ neurons of cPSAP^SERT (% of WT, MFI)
14-day
9-month
****
***
####
F
MW (kDa)
Scramble
PSAP-siRNA
70
55
anti-PSAP
anti-β-actin

Supplementary Fig. 7. Uptake of PSAP in DA and serotonin neurons. (**A**) Representative images of TH (orange), PSAP (magenta), PGRN (cyan) immunofluorescent staining in substantia nigra of WT and $cPSAP^{DAT}$ mice of 4m- and 8m-old. Left panels of each time point are representative low-magnification images; right panels are representative high-magnification images. (**B**) Representative images of TH, PSAP, and PSAP mRNA co-staining in substantia nigra of 8m-old WT and $cPSAP^{DAT}$ mice. (**C**) Representative images of TH (orange), PSAP (magenta), PGRN (cyan) immunofluorescent staining in dorsal raphe nucleus (DRN) of WT and $cPSAP^{SERT}$ mice of 14d- and 9m-old. Left panels of each time point are representative low-magnification images; right panels are representative high-magnification images. (**D-E**) Line graphs showing MFI quantification of PSAP (D) and PGRN (E) in tryptophan hydroxylase (TPH) positive neurons in DRN of WT and $cPSAP^{SERT}$ mice of 14d- and 9m-old. N_{WT} (9d, 13m) = 202, 206 cells from N=3, 3 WT mice respectively, and $N_{cPSAP^{SERT}}$ (9d, 13m) =191, 161 cells from N=3, 3 $cPSAP^{SERT}$ mice respectively; data in (D, E) are normalized to the mean of WT controls. (**F**) Immunoblot of PSAP and β-actin in N2a cells transfected with scramble or PSAP-siRNA. Scale bars, 20 μm. Data are presented as mean ± S.E.M. Two-way ANOVA with Bonferroni's *post hoc* test; * compared to WT, # compared to another time point; ***p<0.001, ****/####p<0.0001.

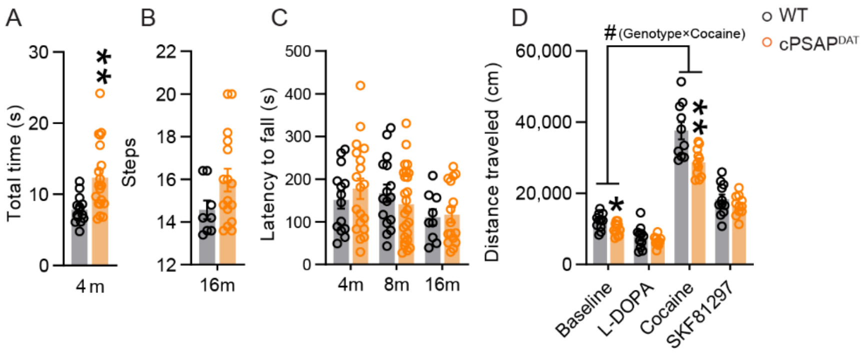

Supplementary Fig. 8. **Behavioral deficiencies and dopaminergic stimulant responsiveness of cPSAP^{DAT} mice.** (**A**) Total time in pole test by WT and cPSAP^{DAT} mice of 4m-old. N_{WT} =14, $N_{cPSAP^{DAT}}$ =19. Mann-Whitney test, ***p*<0.01. (**B**) Steps in beam traversal test by WT and cPSAP^{DAT} mice of 16m-old. N_{WT} =8, $N_{cPSAP^{DAT}}$ =16 cPSAP^{DAT}. Student's t-test. (**C**) Latency to fall in accelerating rotarod test by WT and cPSAP^{DAT} mice of 4m-, 8m-, and 16m-old. N_{WT} =14, 16, 9, $N_{cPSAP^{DAT}}$ =19, 25, 16, respectively for three time points. Two-way ANOVA with Bonferroni's *post hoc* test. (**D**) Bar graph representing distance traveled in open field test by 5m- to 9m-old WT and cPSAP^{DAT} mice at baseline, or treated with L-dopa/benserazide (10/7.5 mg/kg), cocaine (30mg/kg), and SKF81297 (2.5mg/kg) respectively; N_{WT}=10-11, $N_{cPSAP^{DAT}}$=11; paired t-test (*) and two-way ANOVA (#) were applied. Data are presented as mean ± S.E.M. Non-significant *p*-values are not labelled, */$^{\#}$*p*<0.05, **p*<0.01.

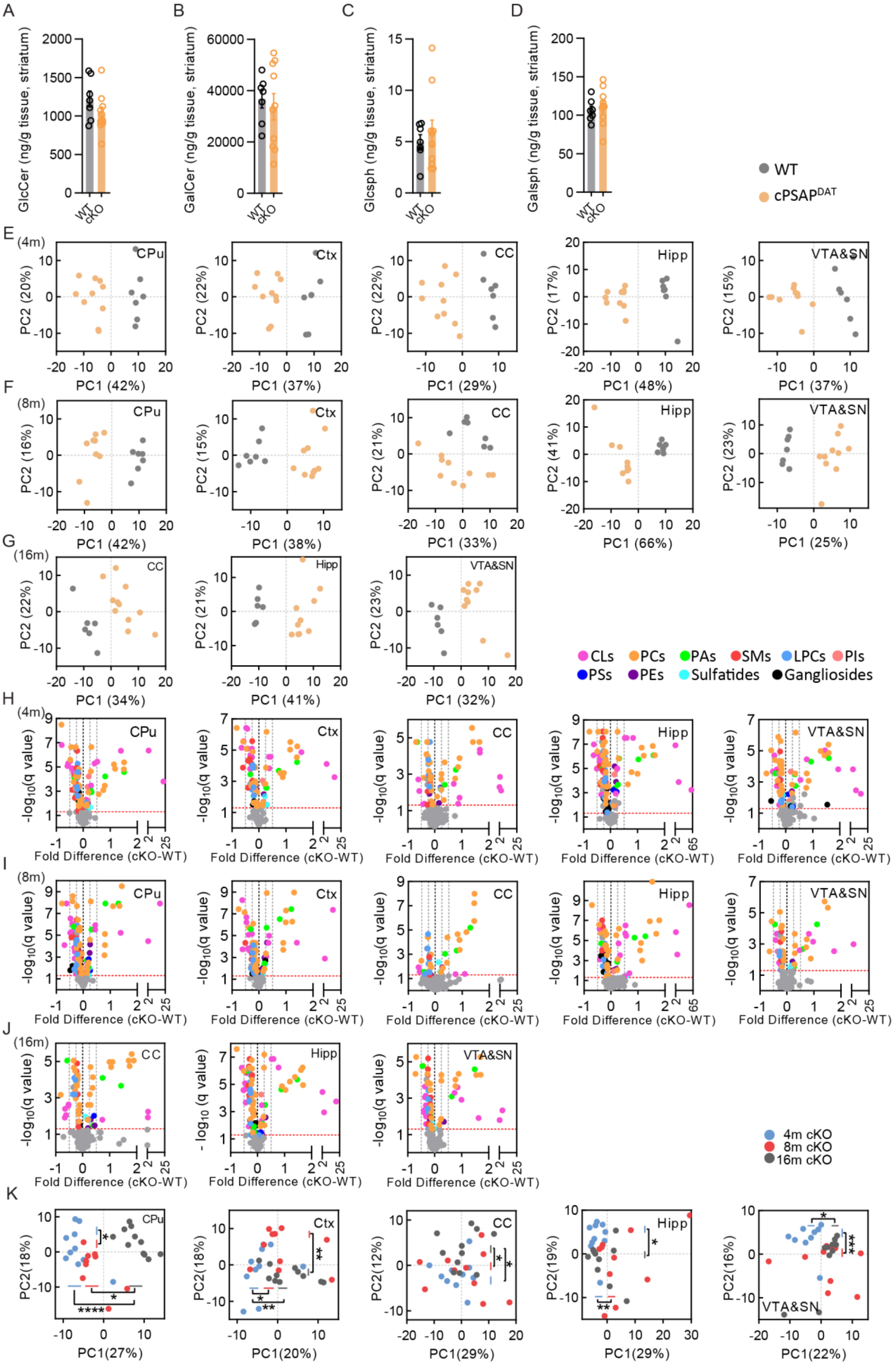

A
B
C
D
E
F
G
H
I
J
K
WT
cPSAPDAT
CLs
PCs
PAs
SMs
LPCs
PIs
PSs
PEs
Sulfatides
Gangliosides
4m cKO
8m cKO
16m cKO
GlcCer (ng/g tissue, striatum)
GalCer (ng/g tissue, striatum)
Glcsph (ng/g tissue, striatum)
Galsph (ng/g tissue, striatum)
(4m)
(8m)
(16m)
CPu
Ctx
CC
Hipp
VTA&SN
PC1 (42%)
PC1 (37%)
PC1 (29%)
PC1 (48%)
PC1 (37%)
PC1 (42%)
PC1 (38%)
PC1 (33%)
PC1 (66%)
PC1 (25%)
PC1 (34%)
PC1 (41%)
PC1 (32%)
-log10(q value)
Fold Difference (cKO-WT)
PC1(27%)
PC1(20%)
PC1(29%)
PC1(29%)
PC1(22%)

Supplementary Fig. 9. Glucosylceramide, galactosylceramide, glucosylsphingosine, and galactosylsphingosine are unaltered in the striatum of cPSAP^{DAT} mice, while lipidomics reveals dramatic and global changes in other lipids in cPSAP^{DAT} mouse brains. (**A-D**) Glucosylceramide (GlcCer) (A), galactosylceramide (GalCer) (B), glucosylsphingosine (GlcCer) (C), and galactosylsphingosine (GalCer) (D) levels in the striatum of WT and cPSAP^{DAT} mouse. Data are presented as mean ± S.E.M. N_{WT} =7, $N_{cPSAP^{DAT}}$ =10. Student's t-test. (**E-G**) Score plots presenting the first and second principal components (PC1 and PC2) generated by the principal component analysis (PCA) of all annotated lipids in different brain regions o©m- (E), 8m- (F), or 16m-old (G) WT (gray) and cPSAP^{DAT} (orange) mice. Each point depicts one mouse. (**H-J**) Volcano plots showing the indicated fold differences and the minus logarithm of q value (-$\log_{10}$ (q value)) of all annotated lipids in different brain regions of two genotypes of 4m- (G), 8m- (I), and 16m-old (H). The red dash line represents FDR (q) = 5%. Lipids regulated with a false discovery rate (FDR, q) < 5% are highlighted with different colors. Each dot depicts one lipid. (**K**) Score plot showing PC1 and PC2 generated by PCA analysis of all annotated lipids in the caudate-putamen (CPu), cortex (Ctx), corpus callosum (CC), hippocampus (Hipp), and ventral tegmental area and substantia nigra (VTA&SN) of 4m- (blue), 8m- (red), and 16m-old (grey) cPSAP^{DAT}. All lipid levels of cPSAP^{DAT} mice are normalized to the mean of corresponding lipid levels of WT mice. Each dot represents one mouse. N=10, 10, 11, respectively. Significant differences among age groups (lines of the same color as dots) in PC1 or PC2 scores are indicated. Kruskal-Wallis test followed by Dunn's *post-hoc* test is applied for PC1 of all brain regions, PC2 of Hipp, and VTA&SN. One-way ANOVA followed by Bonferroni's *post-hoc* test is applied for PC2 of CPu, Ctx, and CC. Non-significant *p* values are not labelled, *p<0.05, **p<0.01, ***p<0.001.

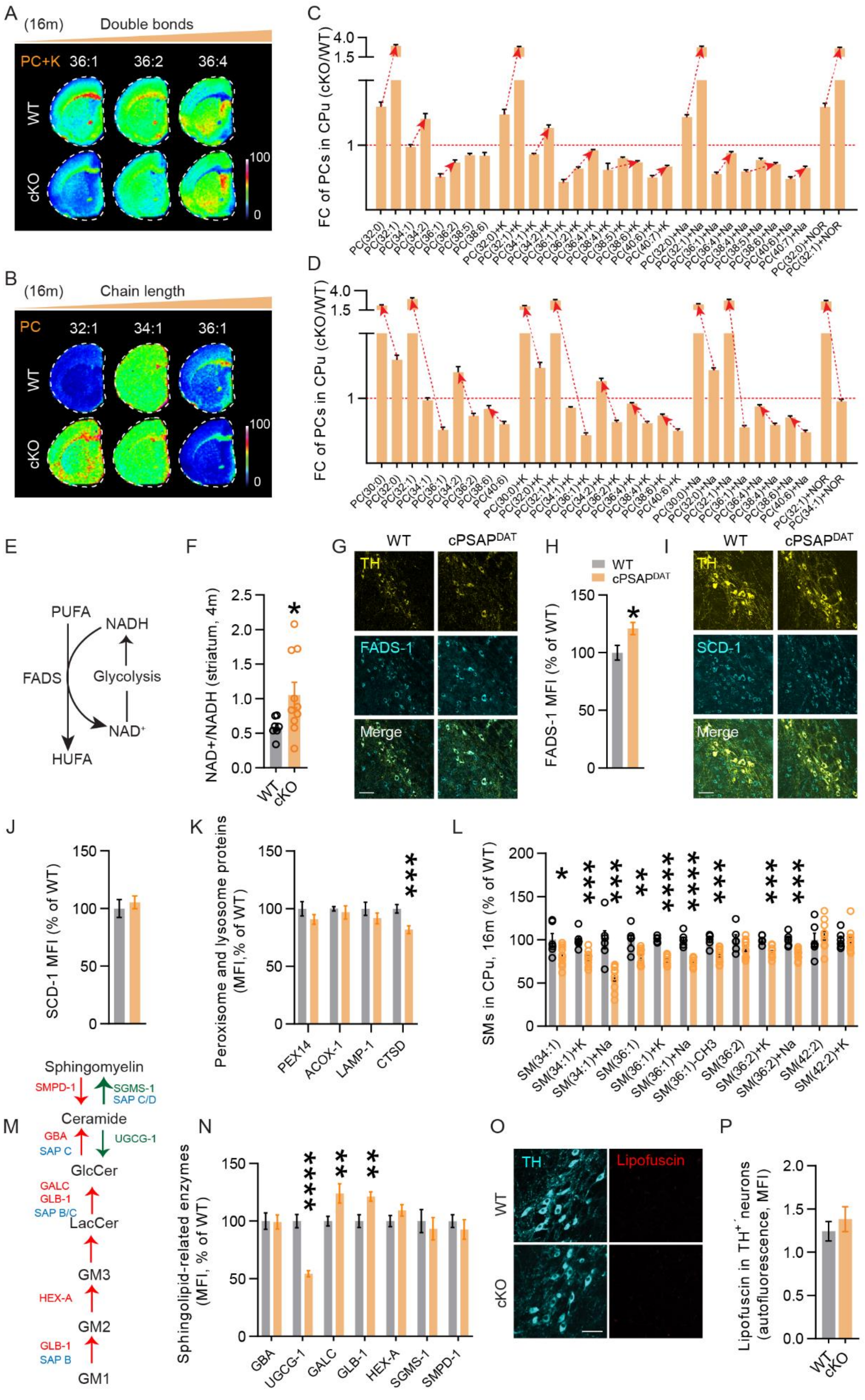
A
(16m)
Double bonds
PC+K
36:1
36:2
36:4
WT
cKO
100
0
B
(16m)
Chain length
PC
32:1
34:1
36:1
C
FC of PCs in CPu (cKO/WT)
D
E
PUFA
NADH
FADS
Glycolysis
NAD+
HUFA
F
NAD+/NADH (striatum, 4m)
G
WT
cPSAPDAT
TH
FADS-1
Merge
H
FADS-1 MFI (% of WT)
I
SCD-1
J
SCD-1 MFI (% of WT)
K
Peroxisome and lysosome proteins (MFI,% of WT)
PEX14
ACOX-1
LAMP-1
CTSD
L
SMs in CPu, 16m (% of WT)
M
Sphingomyelin
SMPD-1
SGMS-1
SAP C/D
Ceramide
GBA
SAP C
UGCG-1
GlcCer
GALC
GLB-1
SAP B/C
LacCer
GM3
HEX-A
GM2
GLB-1
SAP B
GM1
N
Sphingolipid-related enzymes (MFI, % of WT)
GBA
UGCG-1
GALC
GLB-1
HEX-A
SGMS-1
SMPD-1
O
TH
Lipofuscin
P
Lipofuscin in TH+ neurons (autofluorescence, MFI)

Supplementary Fig. 10 cPSAP^{DAT} mice display accumulation of highly unsaturated and shortened lipids along with reduction of sphingolipids throughout the brain, while lipofuscin is unchanged. (**A-B**) Representative ion images of phosphatidylcholine (PC) peaks with chain length and double bonds indicated atop, sorted by numbers of double bonds (A) or chain length (B). (**C-D**) Bar graphs showing PCs arranged by double bonds (C) or chain length (D) with fold changes (FC) indicated. The red dashed arrows denote the accumulation of highly unsaturated (C) and shortened PCs (D). Data are from CPu of 16m-old WT and cPSAP^{DAT} mice. N_{WT} =6, $N_{cPSAP^{©}}$ =11. (**E**) Schematic of glycolytic NAD^{+} recycling by PUFA desaturation. (**F**) NAD^{+} /NADH ratio in the striatum of 4m-old WT and cPSAP^{DAT} mice. N_{WT} =7, $N_{cPSAP^{DAT}}$ =10. Welch's t-test. (**G**) Representative images of TH (yellow) and FADS-1 (cyan) immunofluorescent staining in the SNc of WT and cPSAP^{DAT} mice. (**H**) MFI quantification of FADS-1 in TH positive neurons in the SNc of WT and cPSAP^{DAT} mice; N_{WT} = 59 cells from 4 WT mice, $N_{cPSAP^{DAT}}$ = 78 cells from 4 cPSAP^{DAT} mice. (**I**) Representative images of TH (yellow) and SCD-1 (cyan) immunofluorescent staining in the SNc of WT and cPSAP^{DAT} mice. (**J**) MFI quantification of SCD-1 in TH positive neurons in the SNc of WT and cPSAP^{DAT} mice; N_{WT} = 42 cells from 4 WT mice, $N_{cPSAP^{DAT}}$ = 49 cells from 4 cPSAP^{DAT} mice. (**K**) MFI quantification of PEX14, ACOX-1, LAMP-1, and CTSD in TH positive neurons in the SNc of WT and cPSAP^{DAT} mice; N_{WT} (PEX14, ACOX-1, LAMP-1, CTSD) = 62, 52, 62, 52 cells from 4 WT mice, $N_{cPSAP^{DAT}}$ (PEX14, ACOX-1, LAMP-1, CTSD) = 42, 41, 42, 41 cells from 4 cPSAP^{DAT} mice. (**L**) Quantification of sphingomyelins (SMs) in CPu of 16m-old WT and cPSAP^{DAT} mice. N_{WT} =6, $N_{cPSAP^{DAT}}$ =11. Data are normalized to the means of WT controls. (**M**) Schematic of sphingolipid metabolism with key enzymes and related saposins. (**N**) MFI quantification of GBA, UGCG-1, GALC, GLB-1, HEX-A, SGMS-1, and SMPD-1 in TH positive neurons in the SNc of WT and cPSAP^{DAT} mice; N_{WT} (GBA, UGCG-1, GALC, GLB-1, HEX-A, SGMS-1, SMPD-1) = 52, 40, 52, 53, 60, 33, 60 cells from 4 WT mice, $N_{cPSAP^{DAT}}$ (GBA, UGCG-1, GALC, GLB-1, HEX-A, SGMS-1, SMPD-1) = 53, 52, 48, 44, 48, 28, 56 cells from 4 cPSAP^{DAT} mice. (**O-P**) Representative images (O) and quantification (P) of lipofuscin in TH positive neurons in substantia nigra of 16m-old WT and cPSAP^{DAT} mice. Mean fluorescent intensity (MFI) of autofluorescence is measured. N (WT, cPSAP^{DAT}) =60, 71 cells from N (WT, cPSAP^{DAT}) = 5, 5 mice, respectively. Scale bars, 50μm. MALDI-MS ion images are shown using rainbow scale (scaled to 100% of max ion intensity scale) for visualization. Data are presented as mean ± S.E.M. Student's t-test. *p<0.05, **p<0.01, ***p<0.001, ****p<0.0001.

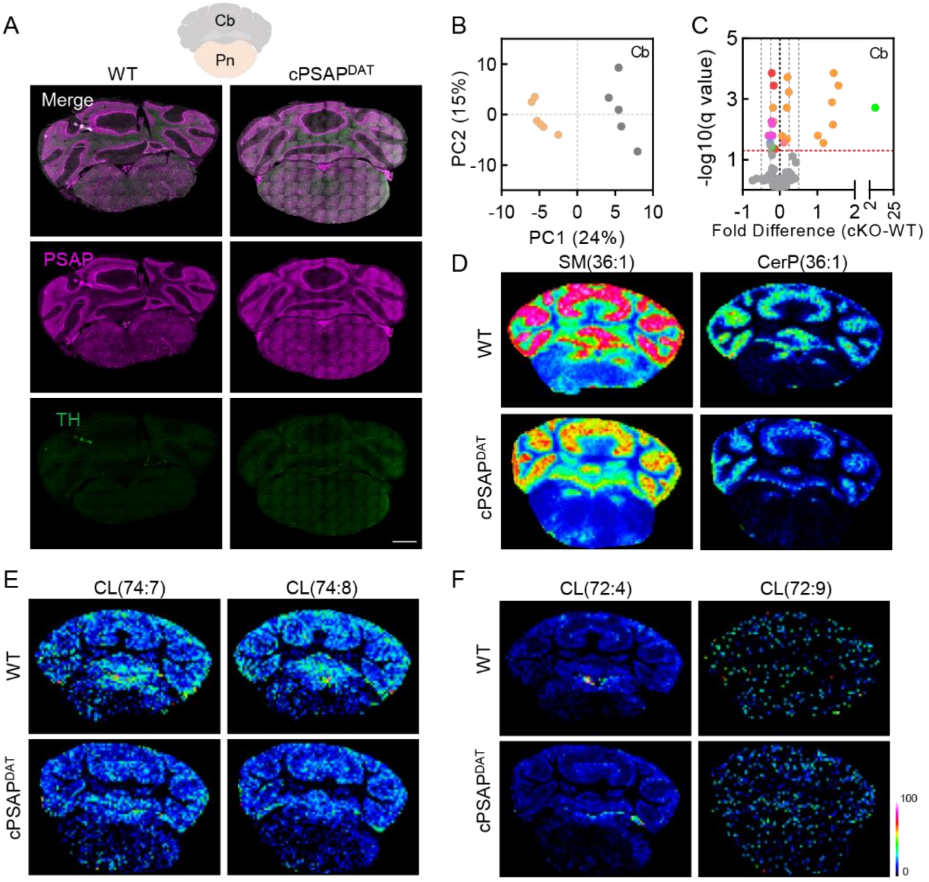

A
Cb
Pn
WT
cPSAP^DAT
Merge
PSAP
TH
B
Cb
PC2 (15%)
PC1 (24%)
C
Cb
-log10(q value)
Fold Difference (cKO-WT)
D
SM(36:1)
CerP(36:1)
WT
cPSAP^DAT
E
CL(74:7)
CL(74:8)
WT
cPSAP^DAT
F
CL(72:4)
CL(72:9)
WT
cPSAP^DAT
100
0

Supplementary Fig. 11. cPSAP^{DAT} mice show similar but less lipid changes in the cerebellum compared to the striatum. (**A**) Representative FISH images of TH mRNA (green) and PSAP mRNA (magenta) in the cerebellum (Cb) of WT and cPSAP^{DAT} mice. Upper, illustration of mouse Cb. Scale bar, 1mm. (**B**) Score plot presenting the PC1 and PC2 generated by the PCA analysis of all annotated lipids in the Cb of 4m-old WT (gray) and cPSAP^{DAT} (orange) mice. Each point depicts one biological replicate. (**C**) Volcano plot showing the indicated fold differences and the minus logarithm of q value ($-\log_{10}$ (q value)) of all detected lipids in the Cb of two genotypes. The red dash line represents FDR (q) = 5%. Lipids regulated with a false discovery rate (FDR, q) < 5% are highlighted with colors. Each dot depicts one lipid. (**D**) Representative ion images of significantly changed sphingolipid examples: SM(36:1) and CerP(36:1) in the Cb of WT and cPSAP^{DAT} mice. (**E**) Representative ion images of significantly changed CL examples: CL(74:7) and CL(74:8) in the Cb of WT and cPSAP^{DAT} mice. (**F**) Representative ion images of unchanged CL examples: CL(72:4) and CL(72:9) in the Cb of WT and cPSAP^{DAT} mice. MALDI-MS ion images are shown using rainbow scale (scaled to 100% of max ion intensity scale) for visualization.

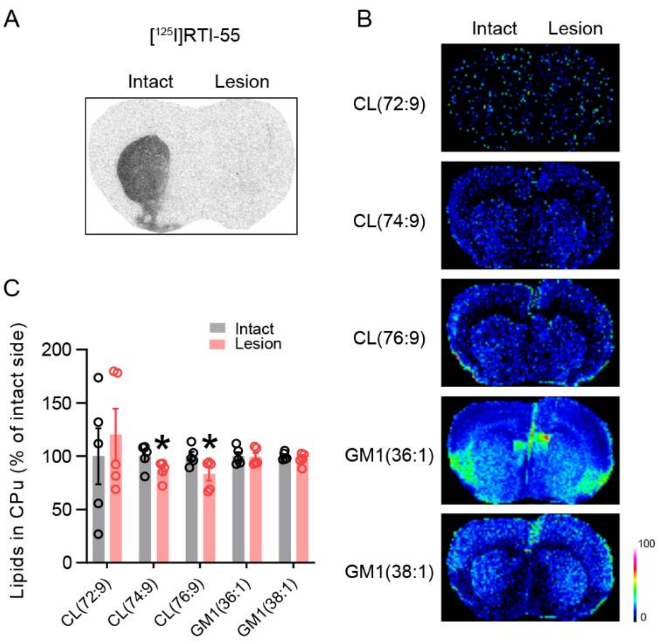

Supplementary Fig. 12. Lipid alterations in the brain of 6-OHDA MFB lesioned WT mice do not resemble those found in cPSAP^{DAT} mice. (**A**) Representative autoradiograph of [^{125}I] RTI-55 binding with DAT on striatal section of WT mice unilaterally lesioned in the medial forebrain bundle (MFB) with 6-OHDA. (**B-C**) Representative ion images (B) of CL(72:9), CL(74:9), CL(76:9), GM1(36:1), and GM1(38:1) in the striatum of lesioned mice and bar graph of lipid quantification in the caudate-putamen (CPu) (C). Each circle represents one mouse; N=5. Data are presented as mean ± S.E.M. Paired student t-tests. * Compared to intact side. *p<0.05. MALDI-MS ion images are shown using rainbow scale (scaled to 100% of max ion intensity scale) for visualization.

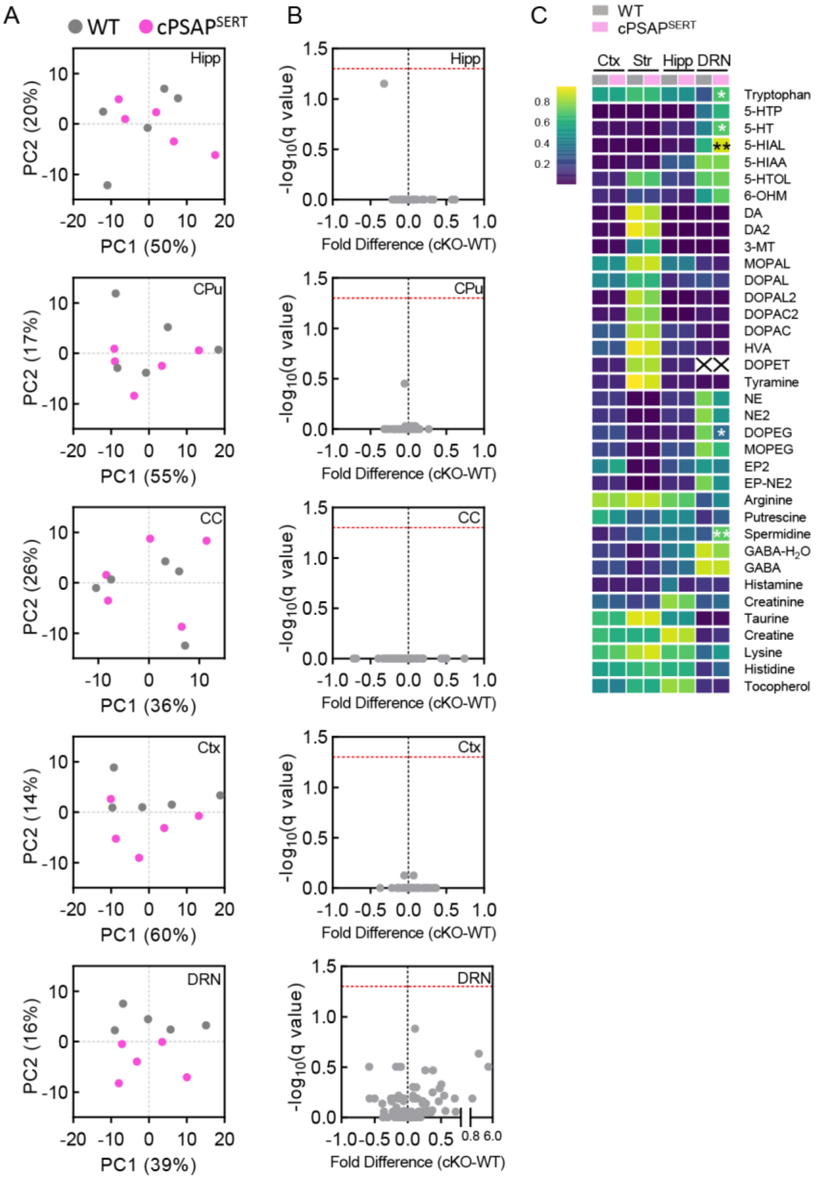
A
WT
cPSAP^SERT
Hipp
CPu
CC
Ctx
DRN
PC2 (20%)
PC1 (50%)
PC2 (17%)
PC1 (55%)
PC2 (26%)
PC1 (36%)
PC2 (14%)
PC1 (60%)
PC2 (16%)
PC1 (39%)
B
-log10(q value)
Fold Difference (cKO-WT)
C
WT
cPSAP^SERT
Ctx
Str
Hipp
DRN
0.8
0.6
0.4
0.2
Tryptophan
5-HTP
5-HT
5-HIAL
5-HIAA
5-HTOL
6-OHM
DA
DA2
3-MT
MOPAL
DOPAL
DOPAL2
DOPAC2
DOPAC
HVA
DOPET
Tyramine
NE
NE2
DOPEG
MOPEG
EP2
EP-NE2
Arginine
Putrescine
Spermidine
GABA-H2O
GABA
Histamine
Creatinine
Taurine
Creatine
Lysine
Histidine
Tocopherol

Supplementary Fig. 13. cPSAP^{SERT} mice display confined accumulation of gangliosides and increased tryptophan metabolism in the dorsal raphe nucleus. (**A**) Score plots presenting PC1 and PC2 derived from PCA analysis of all annotated lipids in different brain regions of 8m-old WT (grey) and cPSAP^{SERT} (magenta) mice. Each point depicts one biological replicate. (**B**) Volcano plots showing the indicated fold differences and -$\log_{10}$ (q value) of all detected annotated lipids in different brain regions of two genotypes. The red dash line represents FDR (q) = 5%. Each dot depicts one lipid. (**C**) Heat map of neurotransmitters and their metabolites along with other detected molecules in different brain regions of two genotypes. Each square represents mean of the corresponding item in the brain regions of the group. For each item, the value is normalized across different brain regions. Student's t-test. Each circle represents one mouse. Data are presented as mean ± S.E.M. *$p<0.05$, **$p<0.01$. Hipp, hippocampus; CPu, caudate-putamen; CC, corpus callosum; Ctx, cortex.

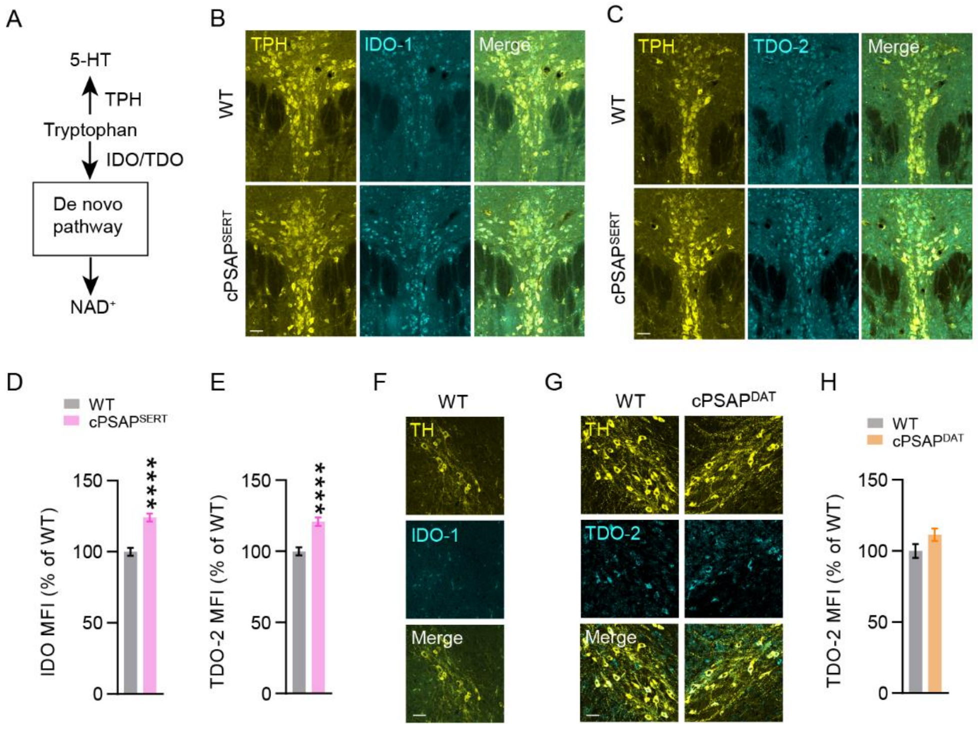

Supplementary Fig. 14. *De novo* pathway of NAD^{+} synthesis is enhanced in the DRN of cPSAP^{SERT} mice, but not in the SNc of cPSAP^{DAT} mice. (**A**) Schematic of NAD^{+} *de novo* biosynthesis from tryptophan. (**B-C**) Representative images of tryptophan hydroxylase (TPH) (yellow) and indoleamine 2,3-dioxygenase 1 (B) or tryptophan 2,3-dioxygenase (C) (IDO-1/TDO-2) (cyan) immunofluorescent staining in the DRN of WT and cPSAP^{SERT} mice. (**D-E**) MFI quantification of IDO-1 (D) and TDO-2 (E) in TPH positive neurons in the DRN of WT and cPSAP^{SERT} mice; N_{WT} (IDO-1, TDO-2) = 281, 273 cells from 3 WT mice, $N_{cPSAP^{SERT}}$ (IDO-1, TDO-2) = 274, 275 cells from 3 cPSAP^{SERT} mice. (**F**) Representative images of TH (yellow) and IDO-1 (cyan) immunofluorescent staining in the SNc of WT mice. (**G**) Representative images of TH (yellow) and TDO-2 (cyan) immunofluorescent staining in the SNc of WT and cPSAP^{DAT} mice. (**H**) MFI quantification of TDO-2 in TH positive neurons in the SNc of WT and cPSAP^{DAT} mice; N_{WT} = 72 cells from 4 WT mice, $N_{cPSAP^{DAT}}$ = 65 cells from 4 cPSAP^{DAT} mice. Scale bars, 50μm. Data are presented as mean ± S.E.M. Student's t-test. *****p*<0.0001.

A

	AAVs injected in SNc			
WT	AAV-α-syn	AAV-α-syn+AAV-PSAP	AAV-GFP	AAV-GFP+AAV-PSAP
cKO	AAV-α-syn	AAV-α-syn+AAV-PSAP	AAV-GFP	AAV-GFP+AAV-PSAP

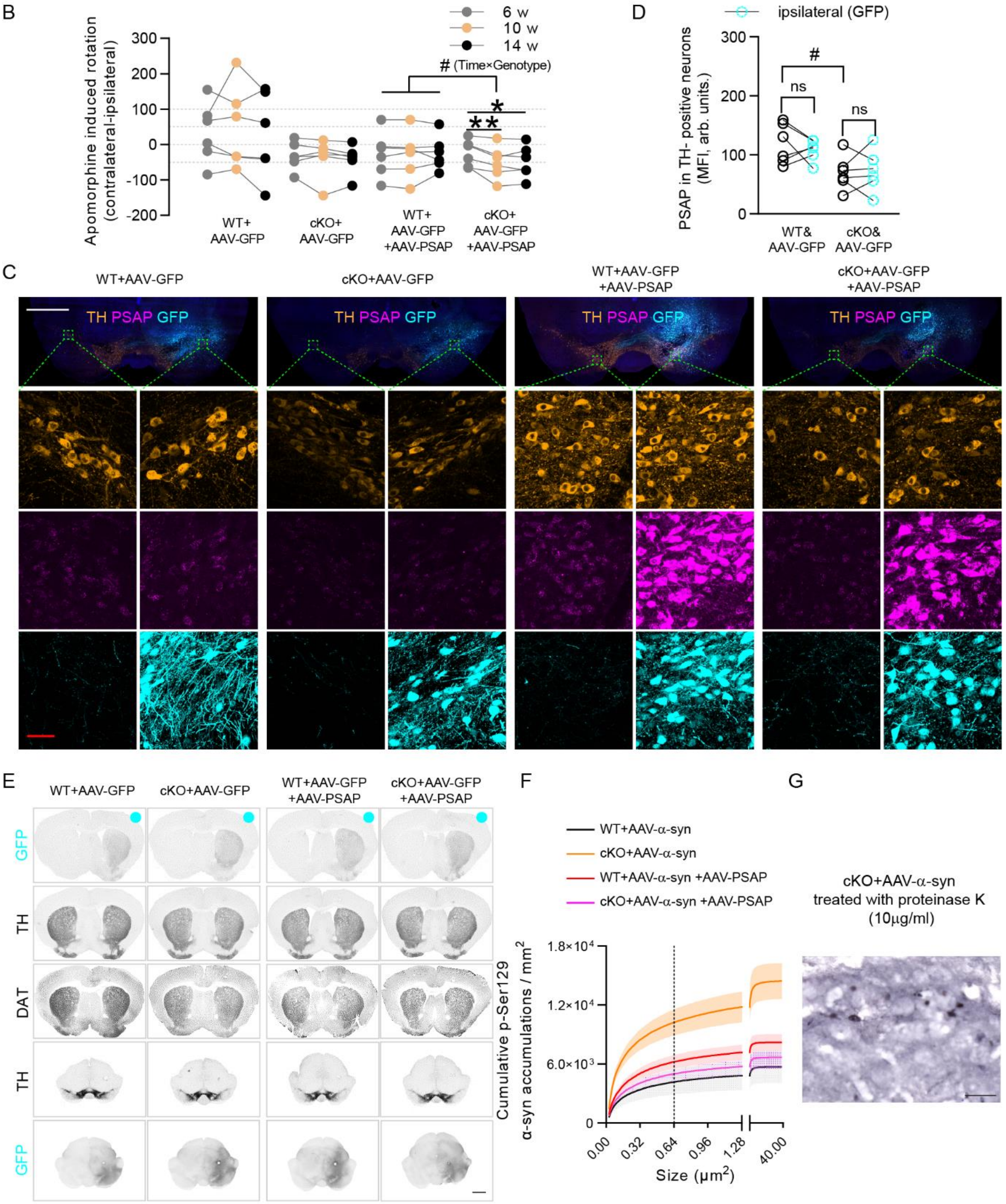

Supplementary Fig. 15. AAV-GFP has no influence on rotational behavior or PSAP levels of DA neurons in substantia nigra of mice, while AAV-PSAP improves rotational behavior of AAV-GFP-injected cPSAP^{DAT} mice. (**A**) Experiment groups of AAV injection (**B**) Quantification of apomorphine-induced net contralateral rotation (contralateral-ipsilateral) of AAV-GFP injected mice with or without AAV-PSAP at 6w (gray), 10w (orange), and 14w (black). Each dot depicts one mouse. N=6 mice in each group. Repeated measures (RM) two-way ANOVA with Bonferroni's *post hoc* test was applied; *compared to 6w, $^{\#}$ interaction. (**C**) Representative images of TH (orange), PSAP (magenta), and GFP (cyan) immunofluorescent staining on postmortem substantia nigra sections of AAV-GFP-injected mice with or without AAV-PSAP. Top panel, low-magnification (scale bar=1000 µm) images of the whole substantia nigra. Bottom panels, high-magnification (scale bar=100 µm) images of TH neurons. (**D**) Quantification of mean fluorescence intensity (MFI) of PSAP staining in TH-positive neurons of AAV-GFP-injected WT and cPSAP^{DAT} mice. Black and cyan circles depict contralateral and ipsilateral PSAP immunoreactivity, respectively. N=6 in each group. RM two-way ANOVA with Bonferroni's *post hoc* test was applied; ns (not significant): compared to contralateral, $^{\#}$ compared to cPSAP^{DAT}. (**E**) Representative images of GFP (cyan dots indicate the injection side) and TH immunohistochemical staining in striatal and substantia nigra sections and DAT staining in striatal sections of AAV-GFP-injected mice. Scale bar, 1mm. (**F**) Quantification of number of p-Ser129 α-syn accumulations of all sizes in striatal sections of AAV-α-syn-injected mice; N=8, 8, 7, 7 mice in four groups, respectively; solid line and shadow represent mean of cumulative numbers and S.E.M, respectively. (**G**) Representative image of p-Ser129 α-syn immunohistochemical staining (enhanced by nickel) in proteinase K-treated striatal sections of AAV-α-syn-injected cPSAP^{DAT} mice; scale bar, 25µm.

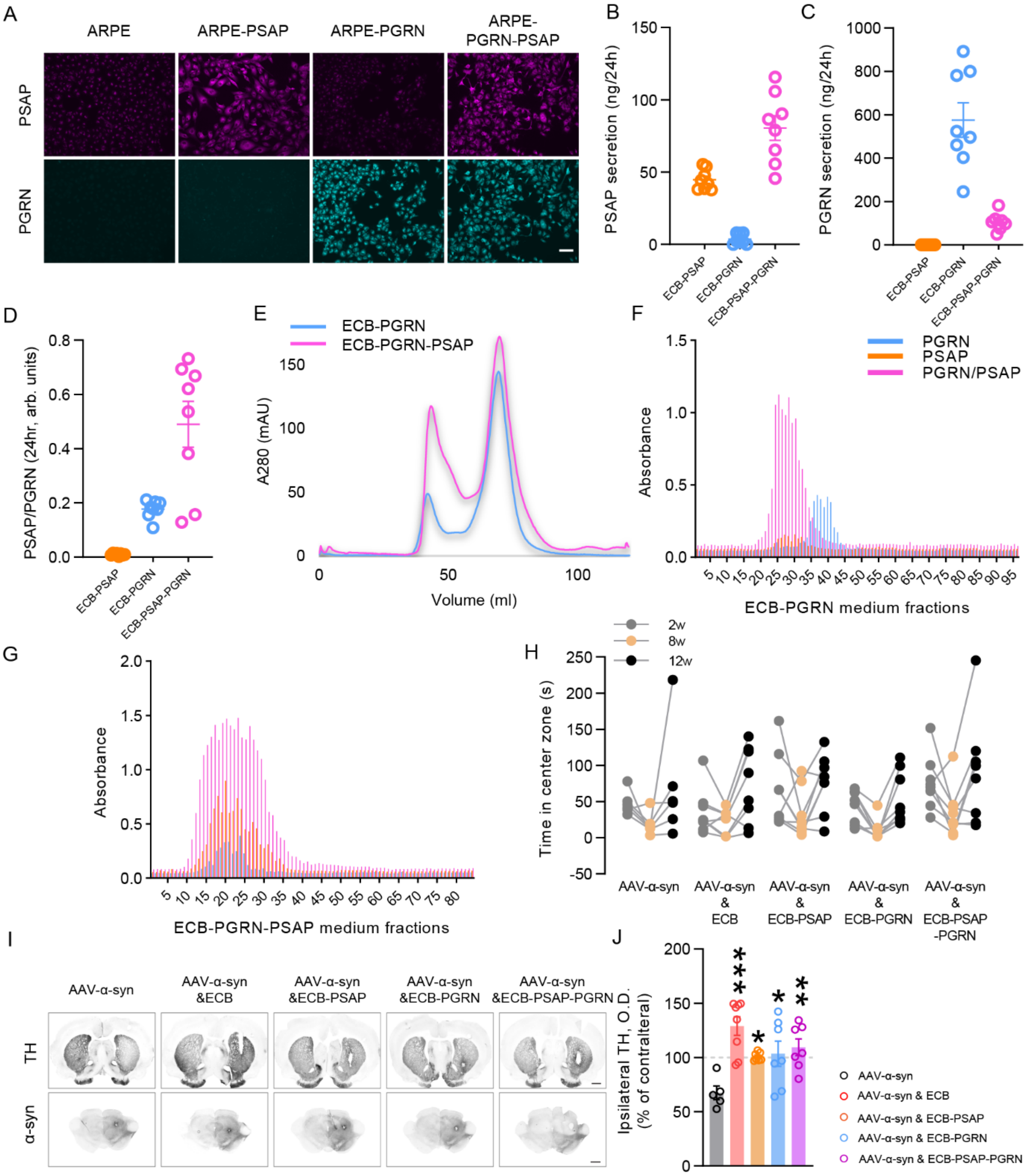
A
ARPE
ARPE-PSAP
ARPE-PGRN
ARPE-PGRN-PSAP
PSAP
PGRN
B
PSAP secretion (ng/24h)
ECB-PSAP
ECB-PGRN
ECB-PSAP-PGRN
C
PGRN secretion (ng/24h)
D
PSAP/PGRN (24hr, arb. units)
E
ECB-PGRN
ECB-PGRN-PSAP
A280 (mAU)
Volume (ml)
F
PGRN
PSAP
PGRN/PSAP
Absorbance
ECB-PGRN medium fractions
G
ECB-PGRN-PSAP medium fractions
H
2w
8w
12w
Time in center zone (s)
AAV-α-syn
AAV-α-syn & ECB
AAV-α-syn & ECB-PSAP
AAV-α-syn & ECB-PGRN
AAV-α-syn & ECB-PSAP-PGRN
I
AAV-α-syn
AAV-α-syn &ECB
AAV-α-syn &ECB-PSAP
AAV-α-syn &ECB-PGRN
AAV-α-syn &ECB-PSAP-PGRN
TH
α-syn
J
Ipsilateral TH, O.D. (% of contralteral)
AAV-α-syn
AAV-α-syn & ECB
AAV-α-syn & ECB-PSAP
AAV-α-syn & ECB-PGRN
AAV-α-syn & ECB-PSAP-PGRN

Supplementary Fig. 16. Secretion capability of ECB devices and their effect on anxiety alleviation and TH preservation of AAV-α-synuclein-injected rats. (**A**) Representative images of PSAP and PGRN immunofluorescent staining on transfected and non-transfected ARPE cells before encapsulation. Scale bar, 25μm. (**B-D**) PSAP (B), PGRN (C), and PSAP/PGRN complex (D) measurements in the culture medium of ECB-PSAP, ECB-PGRN, and ECB-PSAP-PGRN devices, respectively. All devices were cultured for 24h before the measurements. N=8 rats in each group. (**E**) SEC chromatograms from ECB-PGRN (blue) and ECB-PGRN-PSAP (magenta) cells. A280 defines protein-containing fractions. (**F-G**) PGRN, PSAP, and PGRN-PSAP complex ELISAs were conducted on the protein-containing fractions from ECB-PGRN (F) and ECB-PGRN-PSAP (G) cells. Fractions were diluted 4000x, 200x, and 40x for the PGRN, PSAP, and PGRN/PSAP ELISAs, respectively. Data are expressed as absorbance units and not absolute protein levels. (**H**) Quantification of time in the center zone in the open field test of all groups of rats at 2w (gray), 8w (orange), and 12w (black). N=6, 8, 8, 8, 8 rats in five groups, respectively. RM two-way ANOVA with Bonferroni's *post hoc* test was applied. (**I**) Representative images of TH (top panel) and α-syn (bottom panel) immunohistochemical staining in striatal and substantia nigra sections, respectively, of all groups of rats. Scale bar, 1mm. (**J**) Densitometry analysis of ipsilateral TH immunoreactivity in the striatum of all groups of rats. Values are normalized to the mean value of contralateral immunoreactivity. N=6, 8, 7, 7, 7 rats in five groups, respectively. One-way ANOVA with Bonferroni's *post hoc* test was applied. Data in (B-D, G) are presented as mean ± S.E.M. *p<0.05, **p<0.01, ***p<0.001.

4. Supporting Figures

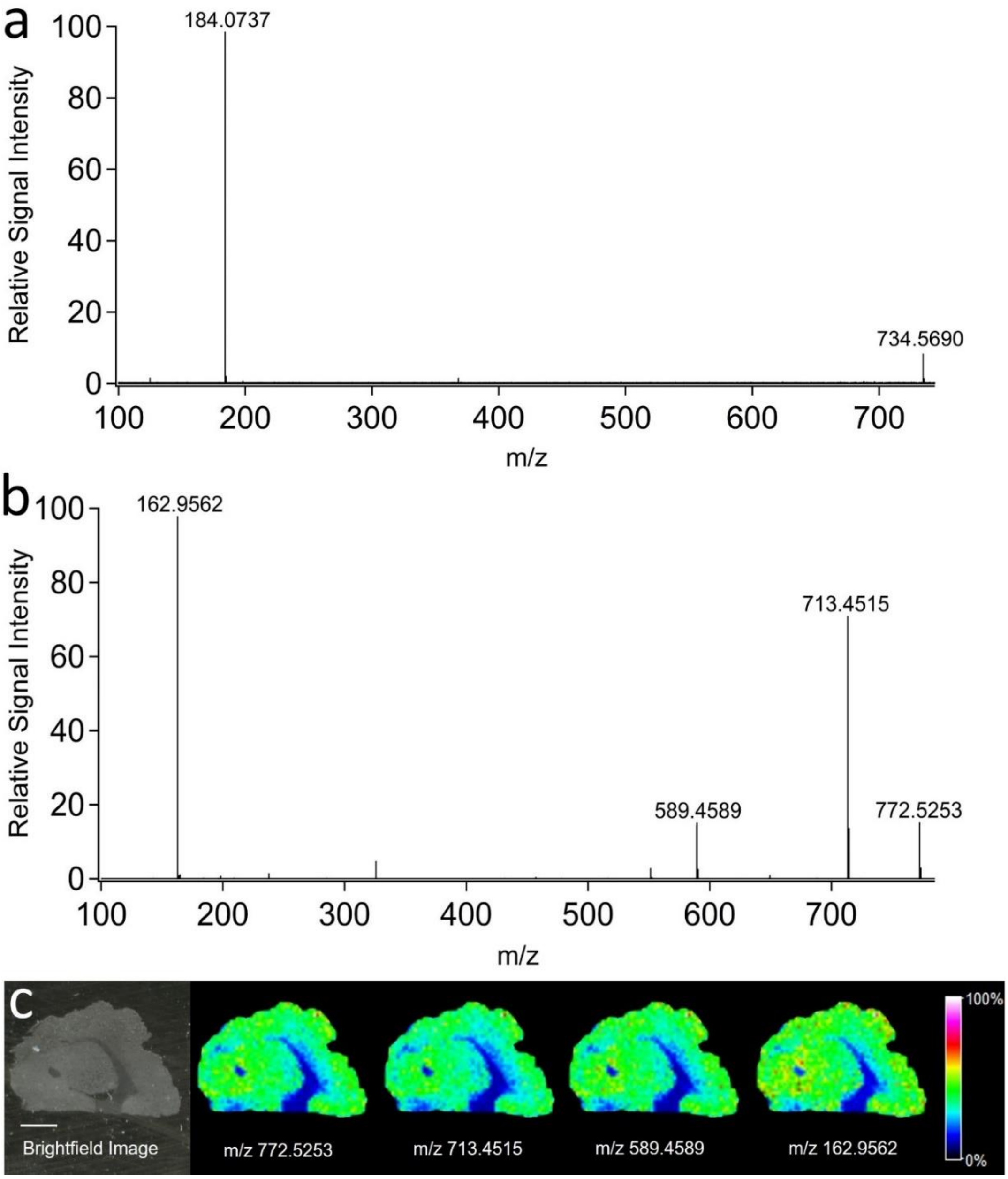

Supplementary Fig. 17. MALDI-MS/MS spectra obtained from mouse brain tissue sections using MALDI-CID-FTICR from the precursor ions at a) *m/z* 734.5690 and b) *m/z* 772.5253. Fragments supporting the assignment of $[PC(32:0)+H]^+$ are found at *m/z* 184.0737 (phosphocholine head group). Fragments supporting the assignment of $[PC(32:0)+K]^+$ are found at *m/z* 713.4515 (loss of trimethylamine), *m/z* 589.4589 (loss of phosphocholine head group) and *m/z* 162.9562 (potassiated cyclophosphane).[1] c) MALDI-MS/MS imaging of coronal mouse brain tissue section using MALDI-CID-FTICR reveals distributions of fragment ions (without normalization) at *m/z* 713.4515 (loss of trimethylamine), *m/z* 589.4589 (loss of phosphocholine head group) and *m/z* 162.9562 (potassiated cyclophosphane) from the precursor ion of $[PC(32:0)+K]^+$. Due to the isolation width of 1 *m/z* additional isobaric and isomeric lipids are potentially also fragmented giving rise to additional product ions. Scale bar in panel (c) is 1000 µm.

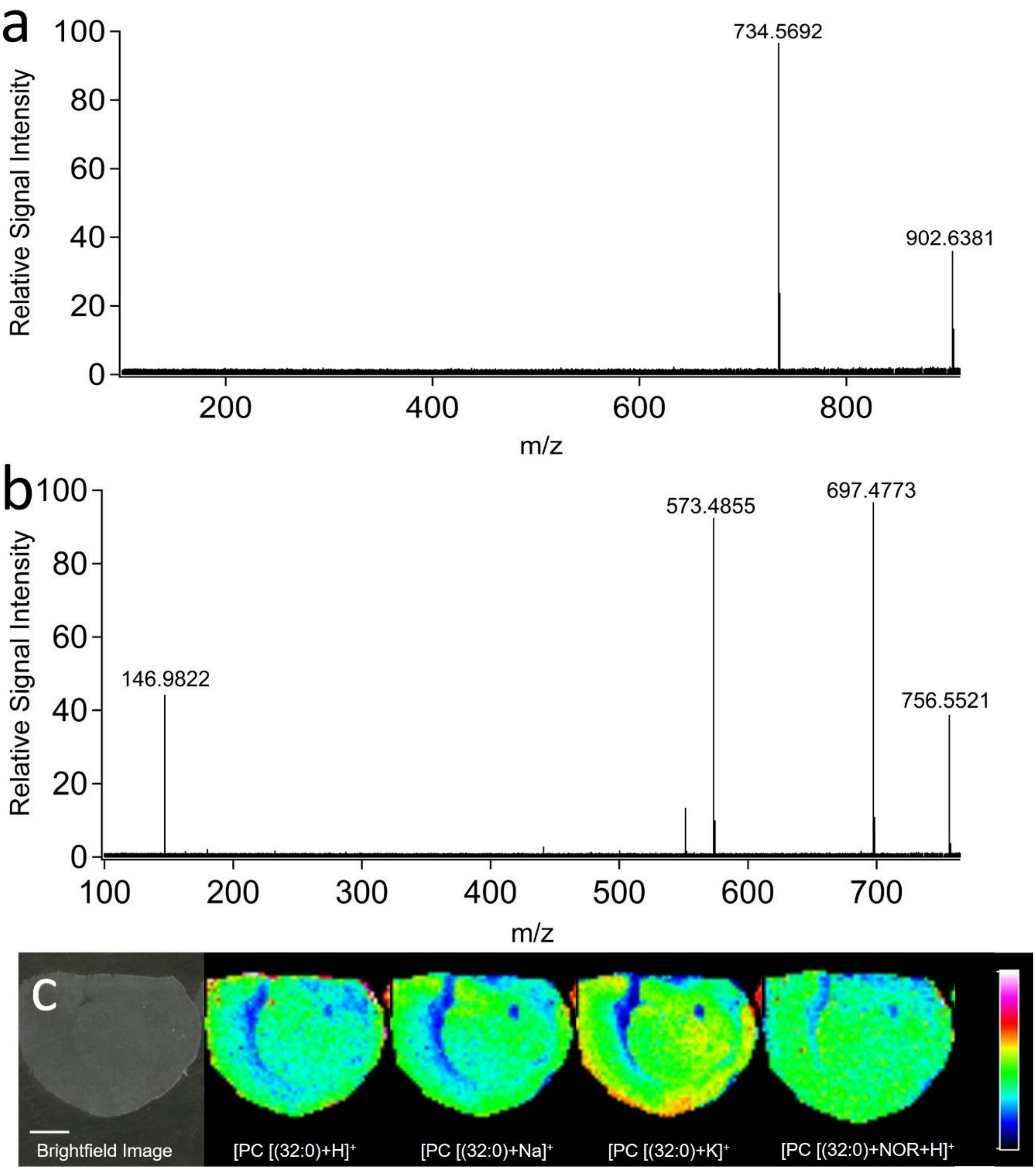

Supplementary Fig. 18. MALDI-MS/MS spectra obtained from mouse brain tissue sections using MALDI-CID-FTICR from the precursor ions at a) *m/z* 902.6381 and b) *m/z* 756.5521. Fragments supporting the assignment of $[PC(32:0)+Norhamane+H]^+$ are found at *m/z* 734.5692 (loss of Norhamane matrix adduct). Fragments supporting the assignment of $[PC(32:0)+Na]^+$ are found at *m/z* 697.4773 (loss of trimethylamine), 573.4855 (loss of phosphocholine head group), and 146.9822 (sodiated cyclophosphane). c) MALDI-FTICR-MSI reveals similar distributions of the ion images (with RMS normalization) of $[PC(32:0)+H]^+$ (intensity scale, 0–70%), $[PC(32:0)+Na]^+$ (intensity scale 0-70%), $[PC(32:0)+K]^+$ (intensity scale (0-100%), $[PC(32:0)+Norharmane+H]^+$(intensity scale 0-70%) on coronal mouse brain tissue section. Due to the isolation width of 1 *m/z* additional isobaric and isomeric lipids are potentially also fragmented giving rise to additional product ions. Scale bar in panel (c) is 1000 µm.

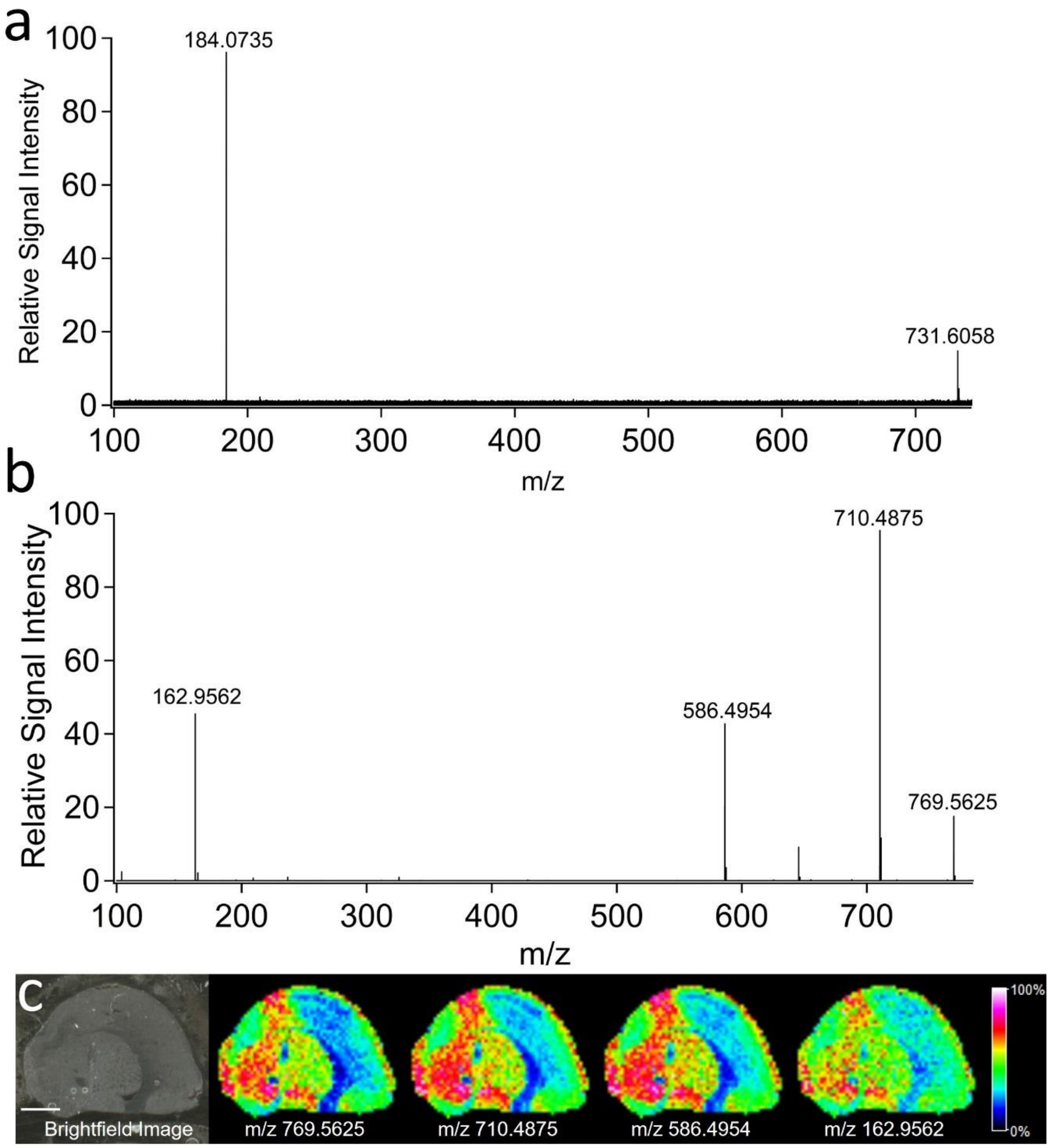

Supplementary Fig. 19. MALDI-MS/MS spectra obtained from mouse brain tissue sections using MALDI-CID-FTICR from the precursor ions at a) *m/z* 731.6058 and b) *m/z* 769.5625. Fragments supporting the assignment of $[SM(36:1)+H]^+$ are found at *m/z* 184.0737 (phosphocholine head group). Fragments supporting the assignment of $[SM(36:1)+K]^+$ are found at *m/z* 710.4875 (loss of trimethylamine), *m/z* 586.4954 (loss of phosphocholine head group) and *m/z* 162.9562 (potassiated cyclophosphane).[1] c) MALDI-MS/MS imaging of coronal mouse brain tissue section using MALDI-CID-FTICR reveals distributions of fragment ions (without normalization) of *m/z* 710.4875 (loss of trimethylamine), *m/z* 586.4954 (loss of phosphocholine head group) and *m/z* 162.9562 (potassiated cyclophosphane) from the precursor ion of $[SM(36:1)+K]^+$. Due to the isolation width of 1 *m/z* additional isobaric and isomeric lipids are potentially also fragmented giving rise to additional product ions. Scale bar in panel (c) is 1000 µm.

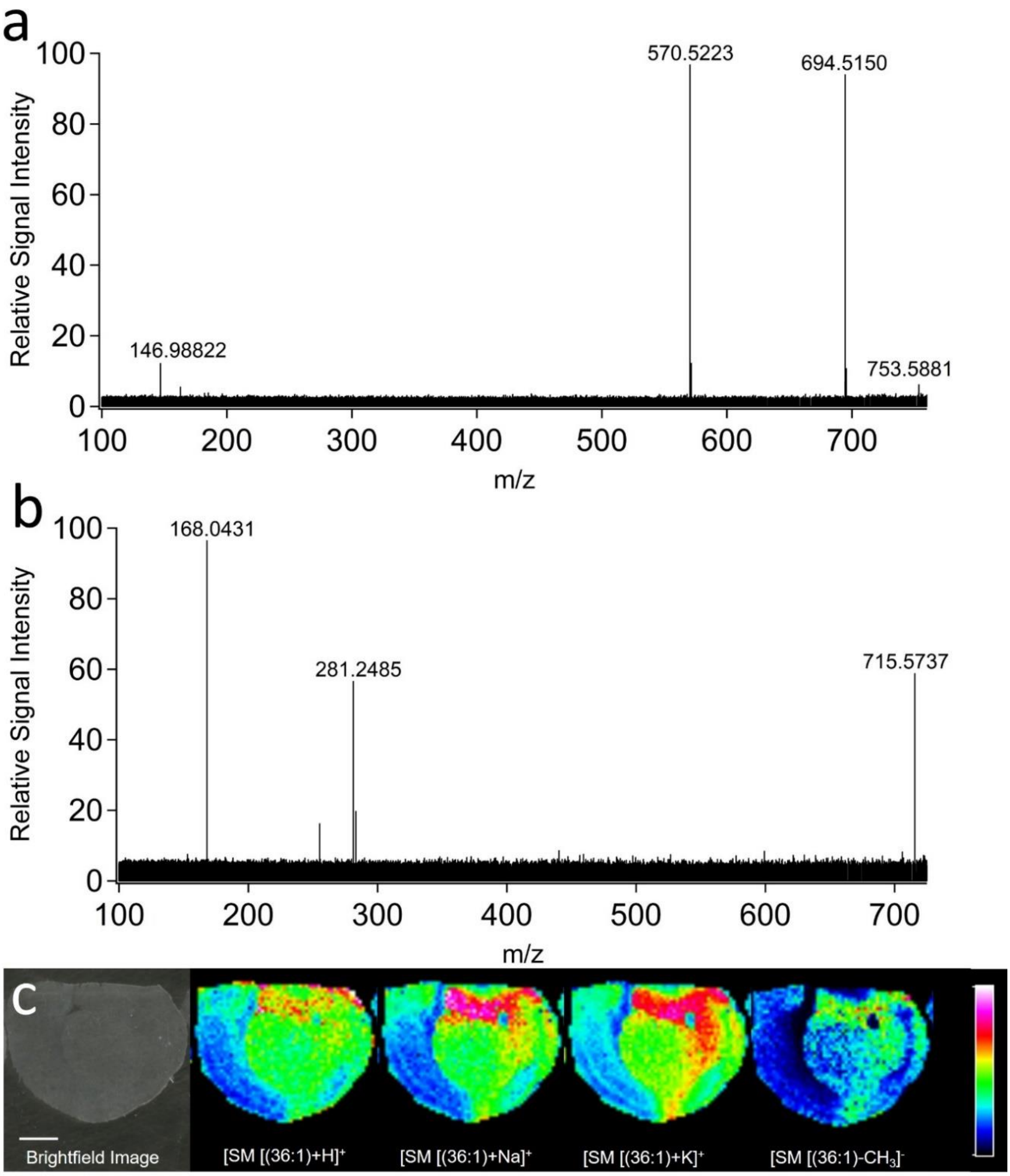

Supplementary Fig. 20. MALDI-MS/MS spectra obtained from mouse brain tissue sections using MALDI-CID-FTICR from the precursor ions at a) *m/z* 753.5881 and b) *m/z* 715.5737. Fragments supporting the assignment of $[PC(36:1)+Na]^+$ are found at *m/z* 694.5150 (loss of trimethylamine), *m/z* 570.2223 (loss of phosphocholine head group), *m/z* 146.9882 (sodiated cyclophosphane). Fragments supporting the assignment of $[SM(36:1)-CH_3]^-$ are found at *m/z* 281.2485 (18:0 fatty acid), *m/z* 168.0431 (loss of methyl from phosphocholine head group). MALDI-FTICR-MSI reveals similar distributions of the ion images (with RMS normalization) of $[SM(36:1)+H]^+$(intensity scale 0-65%), $[SM(36:1)+Na]^+$(intensity scale 0-90%), $[PC(36:1)+K]^+$(intensity scale 0-100%), and $[SM(36:1)-CH_3]^-$ (intensity scale 0-80%) over coronal mouse brain tissue section. Due to the isolation width of 1 *m/z* additional isobaric and isomeric lipids are potentially also fragmented giving rise to additional product ions. Scale bar in panel (c) is 1000 µm.

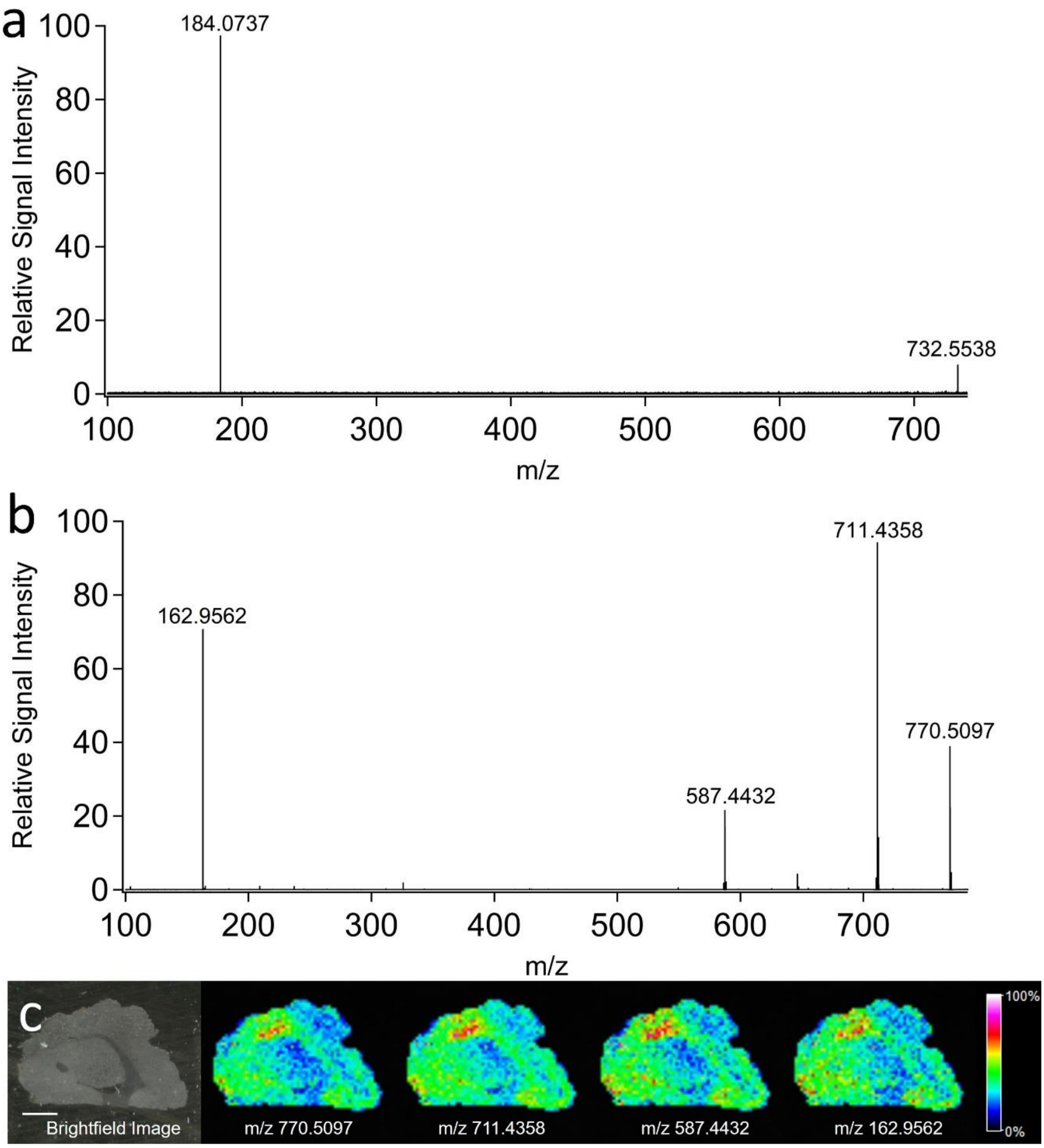

Supplementary Fig. 21. MALDI-MS/MS spectra obtained from mouse brain tissue sections using MALDI-CID-FTICR from the precursor ions at a) *m/z* 732.5538 and b) *m/z* 770.5097. Fragments supporting the assignment of $[PC(32:1)+H]^+$ are found at *m/z* 184.0737 (phosphocholine head group). Fragments supporting the assignment of $[PC(32:1)+K]^+$ are found at *m/z* 711.4358 (loss of trimethylamine), *m/z* 587.4432 (loss of phosphocholine head group) and *m/z* 162.9562 (potassiated cyclophosphane). c) MALDI-MS/MS imaging of coronal mouse brain tissue section using MALDI-CID-FTICR reveals distribution of fragment ions (without normalization) *m/z* 711.4358 (loss of trimethylamine), *m/z* 587.4432 (loss of phosphocholine head group) and *m/z* 162.9562 (potassiated cyclophosphane) from the precursor ion of $[PC(32:1)+K]^+$. Due to the isolation width of 1 *m/z* additional isobaric and isomeric lipids are potentially also fragmented giving rise to additional product ions. Scale bar in panel (c) is 1000 µm.

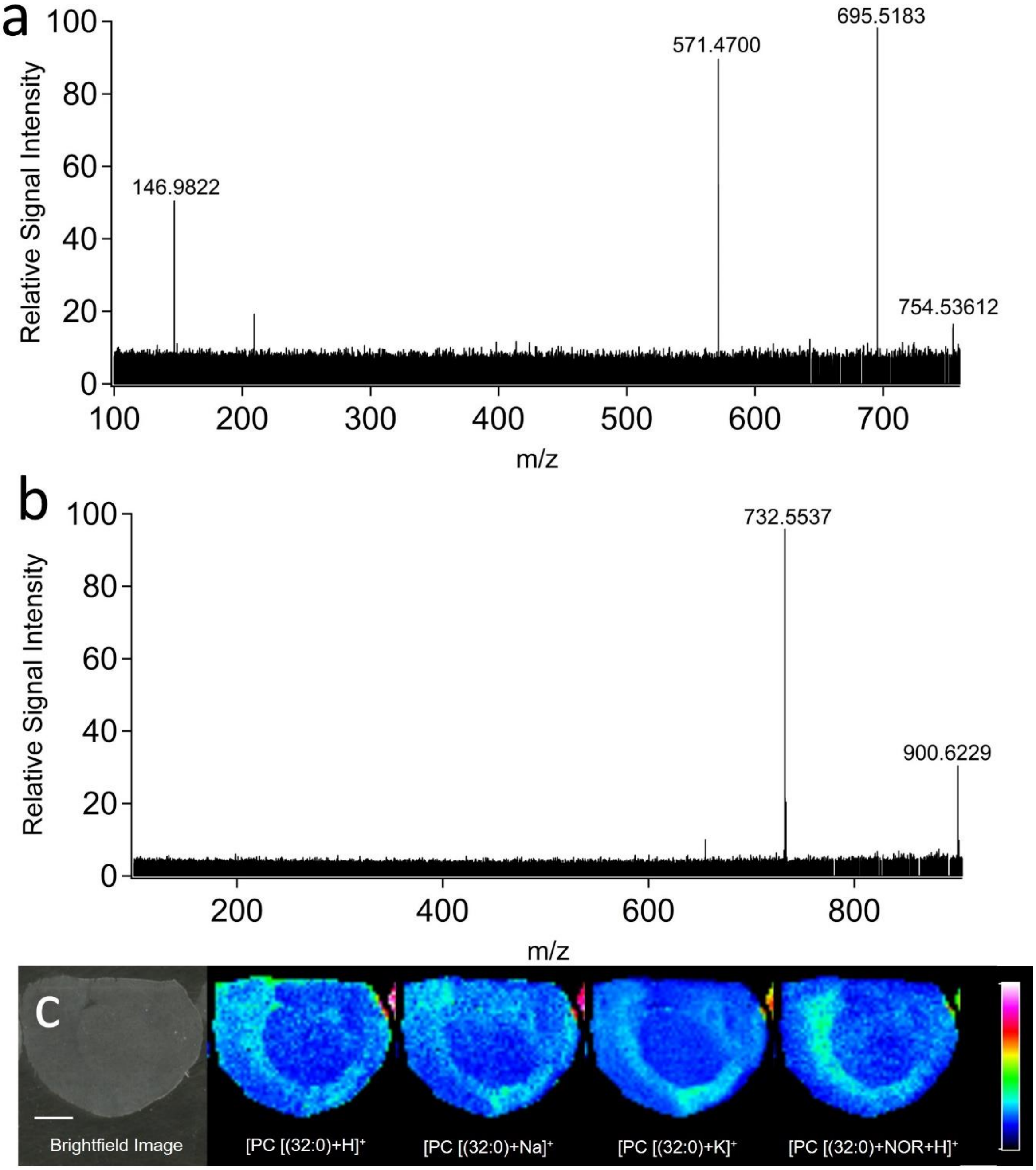

Supplementary Fig. 22. MALDI-MS/MS spectra obtained from mouse brain tissue sections using MALDI-CID-FTICR from the precursor ions at a) *m/z* 754.5361 and b) *m/z* 900.6229. Fragments supporting the assignment of $[PC(32:1)+Na]^+$ are found at *m/z* 695.5183 (loss of trimethylamine), 571.4700 (loss of phosphocholine head group), and 146.9822 (sodiated cyclophosphane). Fragments supporting the assignment of $[PC(32:1)+Norhamane+H]^+$ are found at *m/z* 734.5692 (loss of norhamane matrix adduct). c) MALDI-FTICR-MSI reveals similar distributions of the ion images (with RMS normalization) of $[PC(32:1)+H]^+$(intensity scale 0-70%), $[PC(32:1)+Na]^+$ (intensity scale 0-100%), $[PC(32:1)+K]^+$ (intensity scale 0-100%), $[PC(32:0)+Norharmane+H]^+$(intensity scale 0-80%) over coronal mouse brain tissue section. Due to the isolation width of 1 *m/z* additional isobaric and isomeric lipids are potentially also fragmented giving rise to additional product ions. Scale bar in panel (c) is 1000 µm.

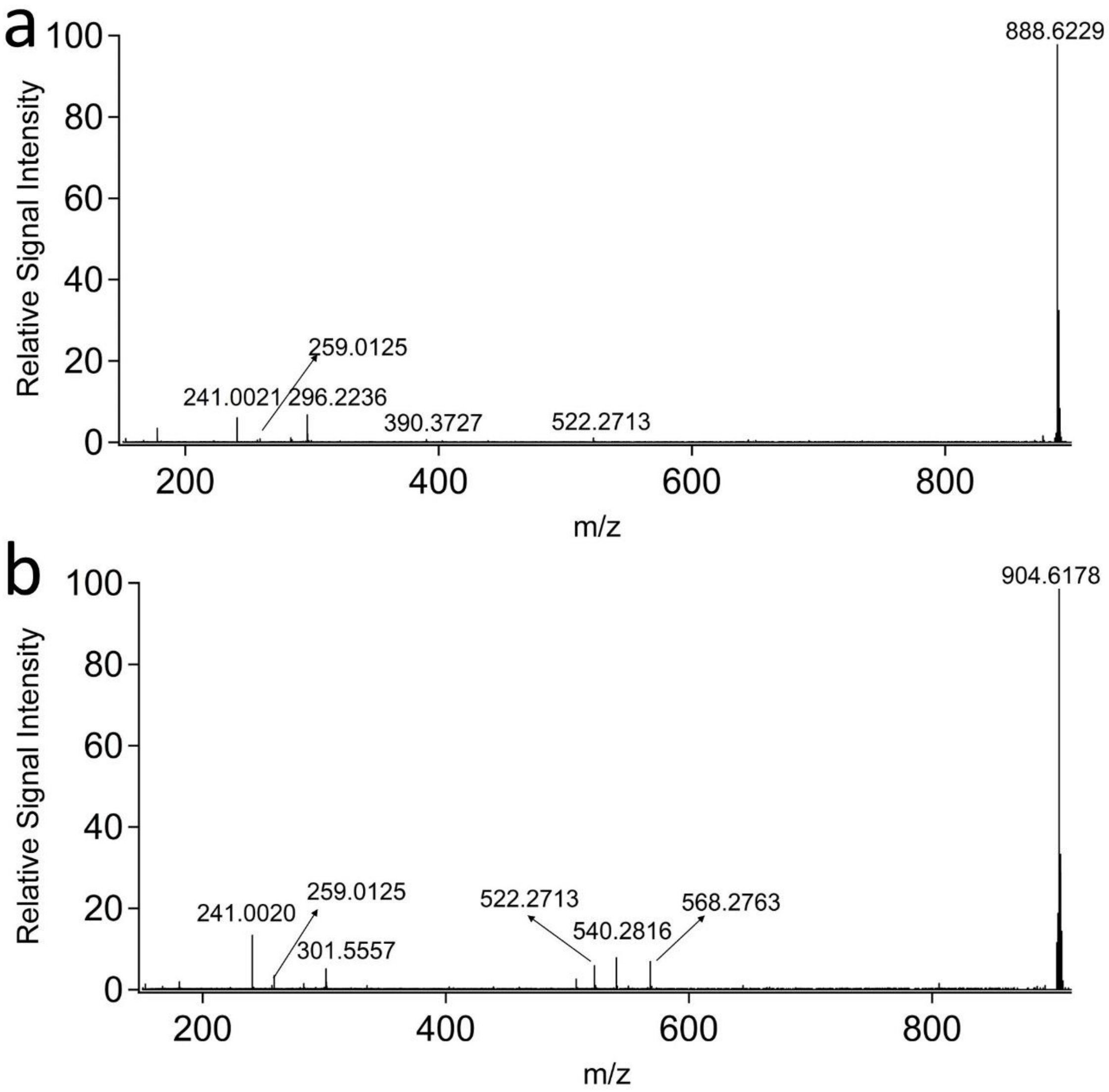

Supplementary Fig. 23. MALDI-MS/MS spectra obtained from mouse brain tissue sections using MALDI-CID-FTICR from the precursor ions at a) *m/z* 888.6229 and b) *m/z* 904.6178. Fragments supporting the assignment of [SHexCer(d42:2)-H]$^-$ are found at *m/z* 241.0021 (loss of water from the sulfated hexose head group), *m/z* 259.0125 (fragment ion of the sulfated hexose head group), *m/z* 522.2713 (consecutive loss of 24:1 fatty acyl and water), *m/z* 390.3727 (related to loss of d18:1 long chain base as $HCOCH=CH(CH_2)_{12}CH_3$).[2] Fragments supporting the assignment of [SHexCer(t42:2)-H]$^-$ are found at *m/z* 540.2816 (loss of 24:1 fatty acyl), *m/z* 522.2713 (consecutive loss of 24:1 fatty acyl and water), *m/z* 241.0021 (loss of water from the sulfated hexose head group), *m/z* 259.0125 (fragment ion of the sulfated hexose head group), *m/z* 568.2763 (the ion resulted from the cleavage of the same OC-CH(OH) bond to expel the fatty acyl moiety as an aldehyde).[2] Due to the isolation width of 1 *m/z* additional isobaric and isomeric lipids are potentially also fragmented giving rise to additional product ions.

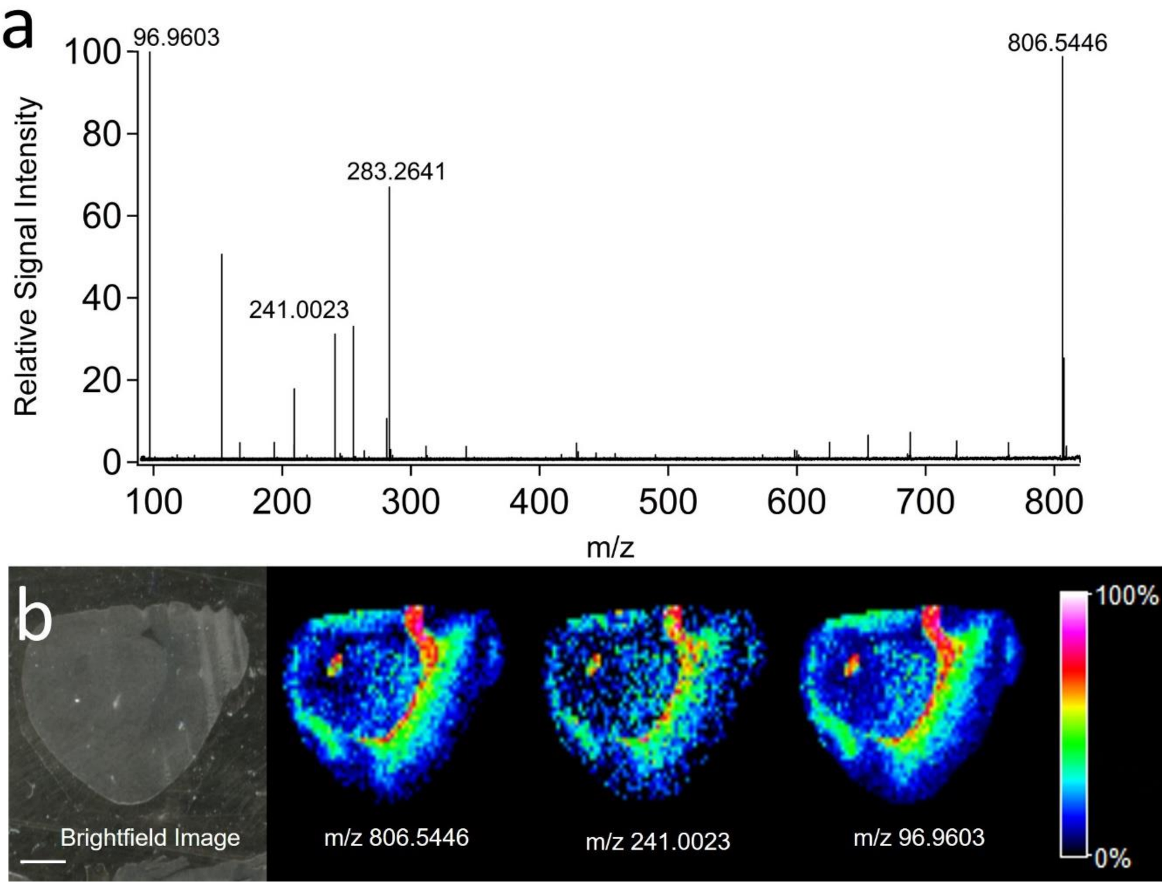

Supplementary Fig. 24. MALDI-MS/MS spectra obtained from mouse brain tissue sections using MALDI-CID-FTICR from the precursor ion at a) *m/z* 806.5446. Fragments supporting the assignment of [SHexCer(d36:1)-H]$^-$ are found at *m/z* 241.0021 (loss of water from the sulfated hexose head group), *m/z* 96.9603 (sulfate ion). b) MALDI-MS/MS imaging of coronal mouse brain tissue section using MALDI-CID-FTICR reveals distributions of fragment ions (without normalization) at *m/z* 241.0021 (loss of water from the sulfated hexose head group), *m/z* 96.9603 (sulfate ion) from the precursor ion of [SHexCer(d36:1)-H]$^-$. Due to the isolation width of 1 *m/z* additional isobaric and isomeric lipids are potentially also fragmented giving rise to additional product ions. Scale bar in panel (b) is 1000 µm.

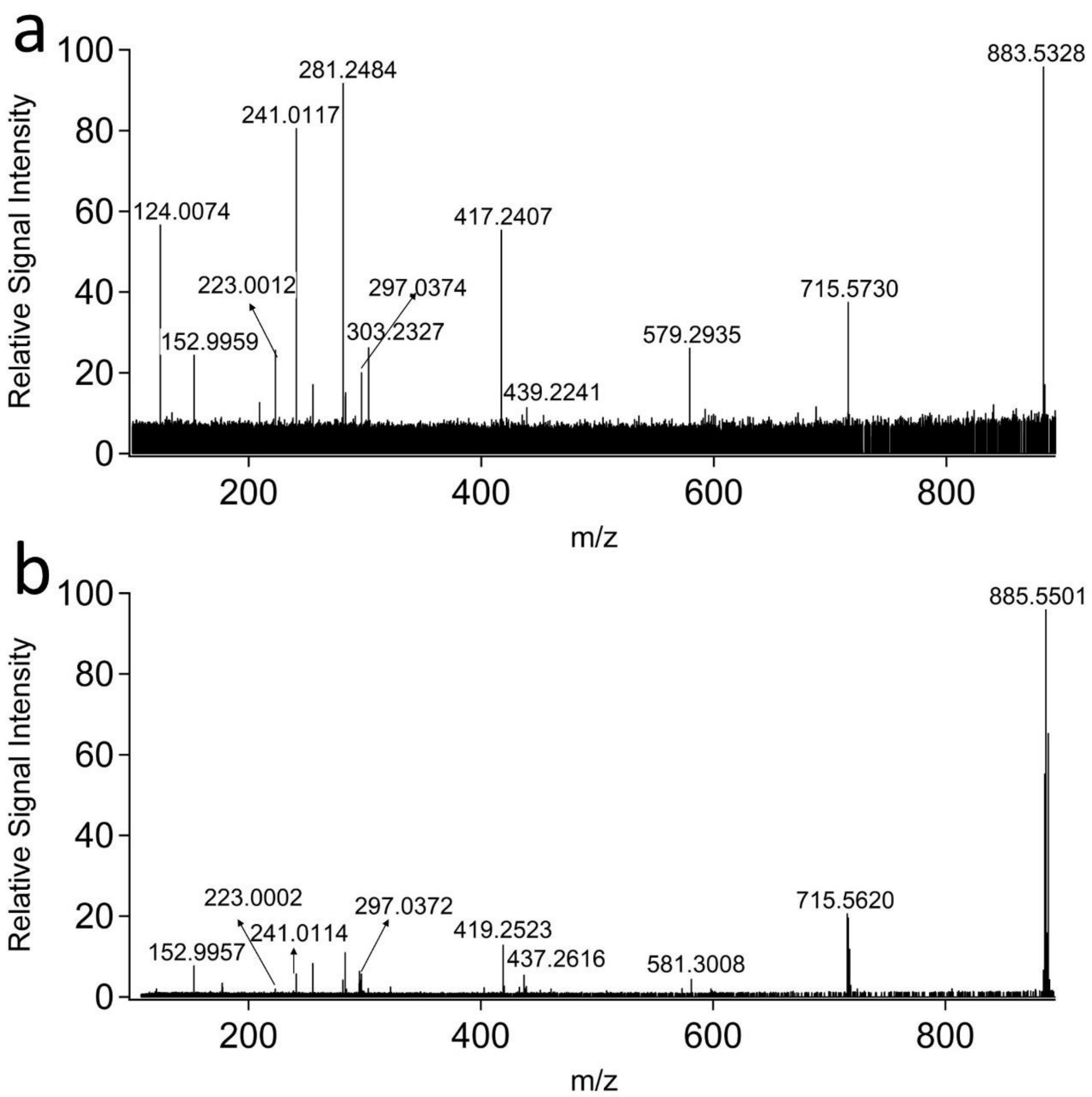

Supplementary Fig. 25. MALDI-MS/MS spectra obtained from mouse brain tissue sections using MALDI-CID-FTICR from the precursor ions at a) *m/z* 883.5328 and b) *m/z* 885.5501. Fragments supporting the assignment of [PI (38:5)-H]$^-$ are found at *m/z* 579.2935 (loss of sn2 acyl chain as ketene (RCH=C=O)), *m/z* 417.2407 (neutral loss of sn2 RCOOH group and inositol), *m/z* 297.0372 (glycerophosphoinositol-2H_2O), *m/z* 281.2484 (sn1 RCOO- ion), *m/z* 223.0012 (inositol phosphate ion-2H_2O), *m/z* 152.9959 (glycerol-3-phosphate ion with loss of H_2O), *m/z* 241.0114 (inositol phosphate ion-H_2O). Fragments supporting the assignment of [PI (38:4)-H]$^-$ are found at *m/z* 581.3008 (loss of sn2 acyl chain as ketene (RCH=C=O)), *m/z* 419.2523 (neutral loss of sn2 RCOOH group and inositol), *m/z* 297.0372 (glycerophosphoinositol-2H_2O), *m/z* 223.0012 (inositol phosphate ion- 2H_2O), *m/z* 152.9959 (glycerol-3-phosphate ion with loss of H_2O). Due to the isolation width of 1 *m/z* additional isobaric and isomeric lipids are potentially also fragmented giving rise to additional product ions.

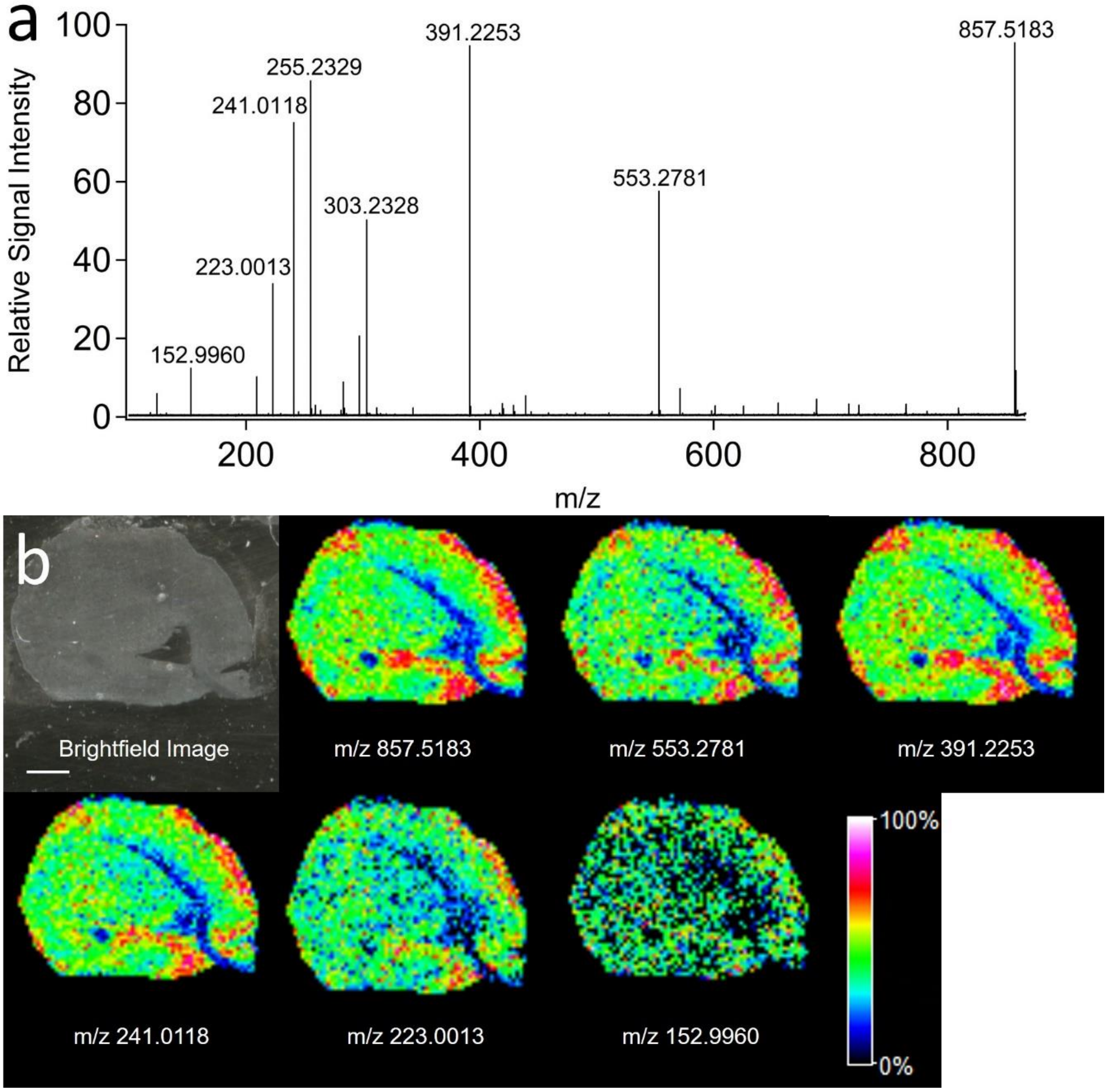

Supplementary Fig. 26. MALDI-MS/MS spectra obtained from mouse brain tissue sections using MALDI-CID-FTICR from the precursor ion at a) *m/z* 857.5183. Fragments supporting the assignment of [PI (36:4)-H]$^-$ are found at *m/z* 553.2781 (neutral loss of sn2 RCOOH group), *m/z* 391.2253 (neutral loss of sn2 RCOOH group and inositol), *m/z* 255.2329 (sn1 RCOO$^-$ ion), 303.2328 (sn2 RCOO$^-$ ion), *m/z* 223.0012 (inositol phosphate ion-2H_2O), *m/z* 152.9959 (glycerol-3-phosphate ion with loss of H_2O), *m/z* 241.0114 (inositol phosphate ion-H_2O). b) MALDI-MS/MS imaging of coronal mouse brain tissue section using MALDI-CID-FTICR reveals distributions of fragment ions (without normalization) at *m/z* 553.2781 (neutral loss of sn2 RCOOH group), *m/z* 391.2253 (neutral loss of sn2 RCOOH group and inositol), *m/z* 223.0012 (inositol phosphate ion-2H_2O), *m/z* 152.9959 (glycerol-3-phosphate ion with loss of H_2O), *m/z* 241.0114 (inositol phosphate ion-H_2O) from the precursor ion of [PI (36:4)-H]$^-$. Due to the isolation width of 1 *m/z* additional isobaric and isomeric lipids are potentially also fragmented giving rise to additional product ions. Scale bar in panel (b) is 1000 µm.

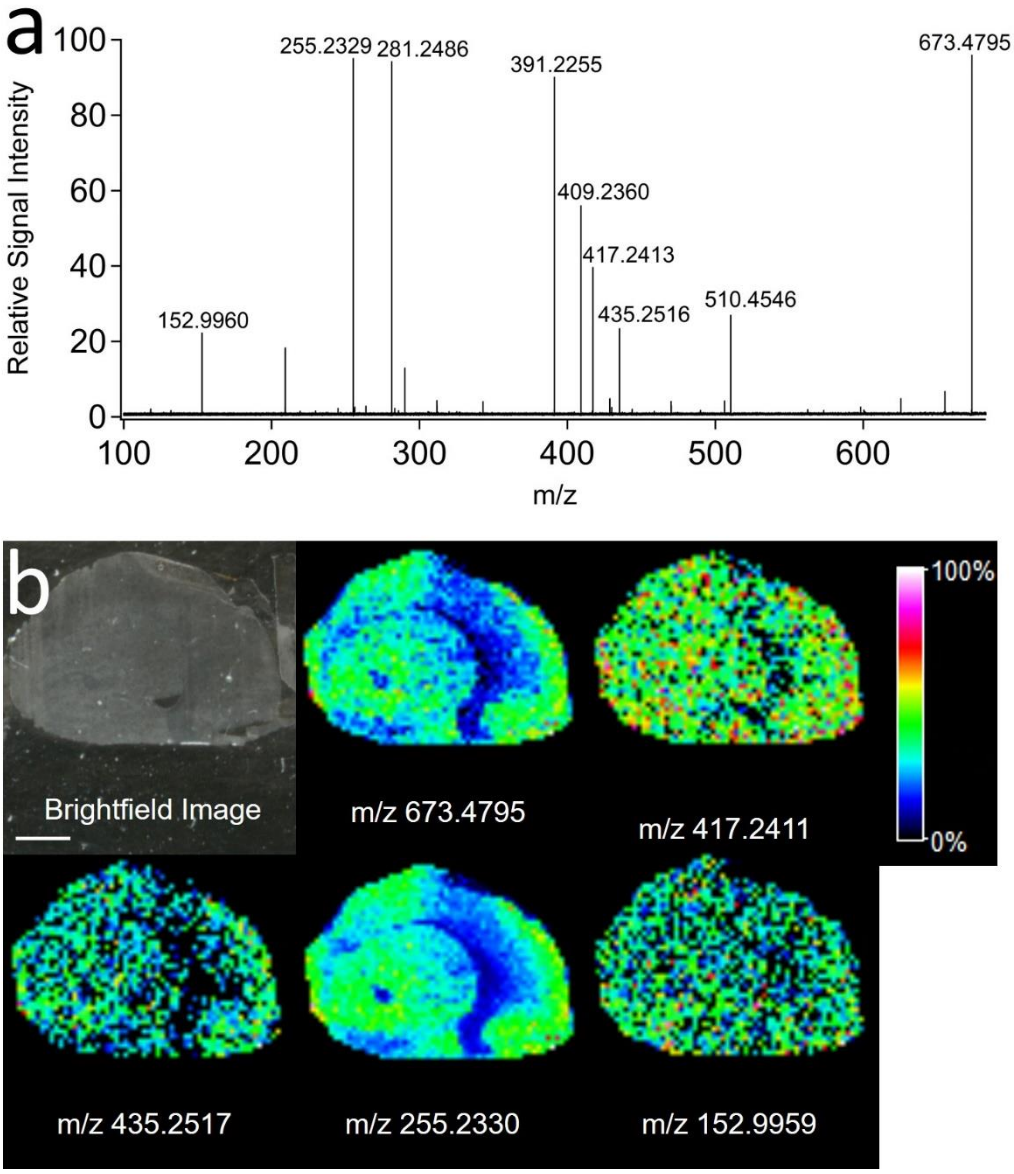

Supplementary Fig. 27. MALDI-MS/MS spectra obtained from mouse brain tissue sections using MALDI-CID-FTICR from the precursor ion at a) *m/z* 673.4795. Fragments supporting the assignment of [PA (34:1)-H]$^-$ are found at *m/z* 435.2516 (loss of sn1 acyl chain as ketene (RCH=C=O)), *m/z* 417.2412 (neutral loss of sn1 RCOOH), *m/z* 409.2360 (loss of sn2 acyl chain as ketene (RCH=C=O)), *m/z* 391.2255 (neutral loss of sn2 RCOOH group, *m/z* 255.2329 (sn1 RCOO$^-$ ion), *m/z* 281.2486 (sn2 RCOO$^-$ ion), *m/z* 152.9960 (glycerol-3-phosphate ion with loss of H_2O). b) MALDI-MS/MS imaging of coronal mouse brain tissue section using MALDI-CID-FTICR reveals distributions of fragment ions (without normalization) at *m/z* 435.2516 (loss of sn1 acyl chain as ketene (RCH=C=O)), *m/z* 417.2412 (neutral loss of sn1 RCOOH), *m/z* 152.9960 (glycerol-3-phosphate ion with loss of H_2O), *m/z* 255.2329 (sn1 RCOO$^-$ ion) from the precursor ion of [PA (34:1)-H]$^-$. Due to the isolation width of 1 *m/z* additional isobaric and isomeric lipids are potentially also fragmented giving rise to additional product ions. Scale bar in panel (b) is 1000 µm.

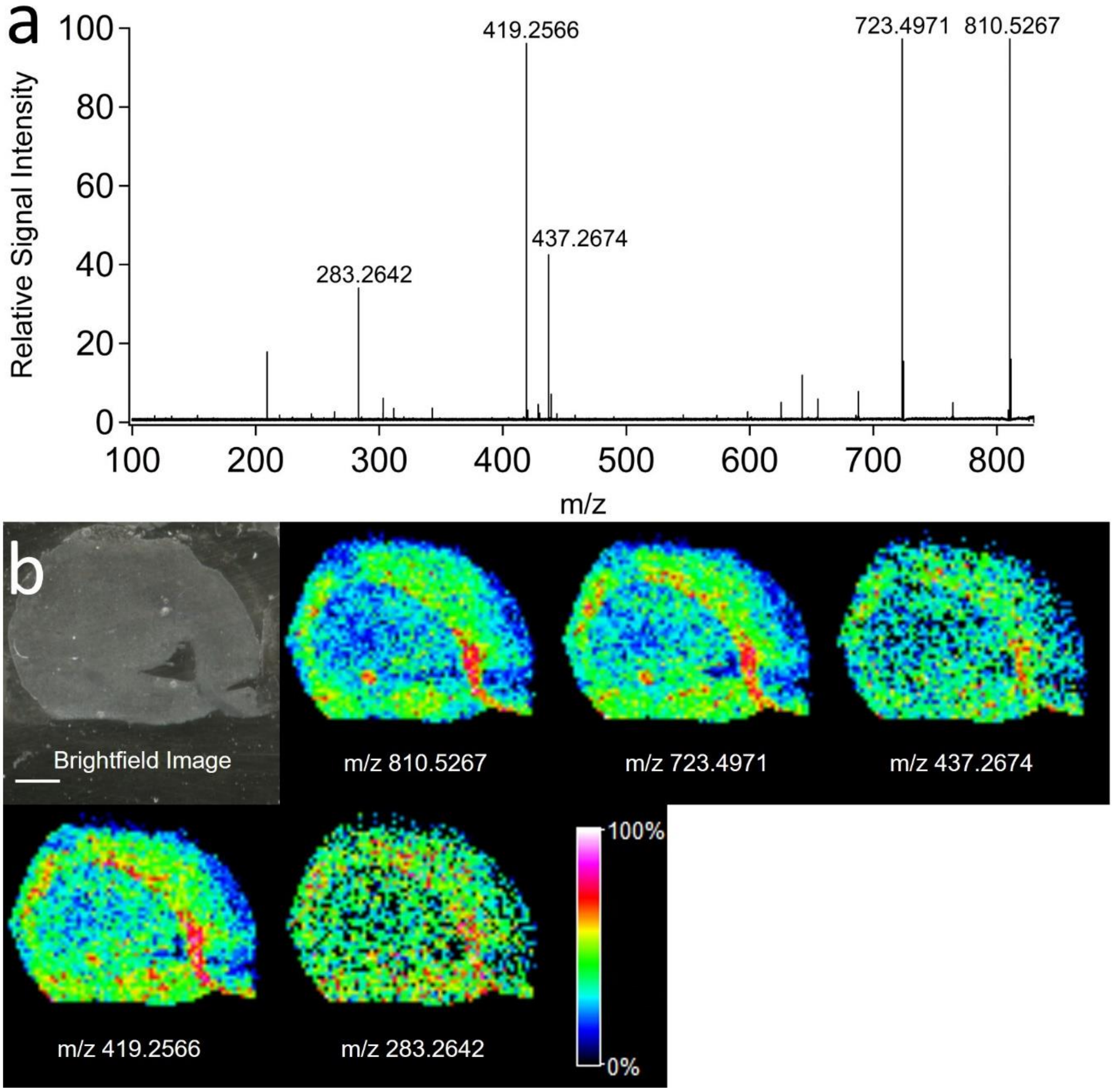

Supplementary Fig. 28. MALDI-MS/MS spectra obtained from mouse brain tissue sections using MALDI-CID-FTICR from the precursor ion at a) *m/z* 810.5267. Fragments supporting the assignment of $[PS(38:4)-H]^-$ are found at *m/z* 723.4971 (loss of serine from precursor ion), *m/z* 437.2674 (loss of sn2 acyl chain as ketene (RCH=C=O) and serine), *m/z* 419.2566 (neutral loss of sn2 RCOOH group and serine, *m/z* 283.2642 (sn1 $RCOO^-$ ion). b) MALDI-MS/MS imaging of coronal mouse brain tissue section using MALDI-CID-FTICR reveals distributions of fragment ions (without normalization) at *m/z* 723.4971 (loss of serine from precursor ion), *m/z* 437.2674 (loss of sn2 acyl chain as ketene (RCH=C=O) and serine), *m/z* 419.2566 (neutral loss of sn2 RCOOH group and serine, *m/z* 283.2642 (sn1 $RCOO^-$ ion) from the precursor ion of $[PS(38:4)-H]^-$. Due to the isolation width of 1 *m/z* additional isobaric and isomeric lipids are potentially also fragmented giving rise to additional product ions. Scale bar in panel (b) is 1000 μm.

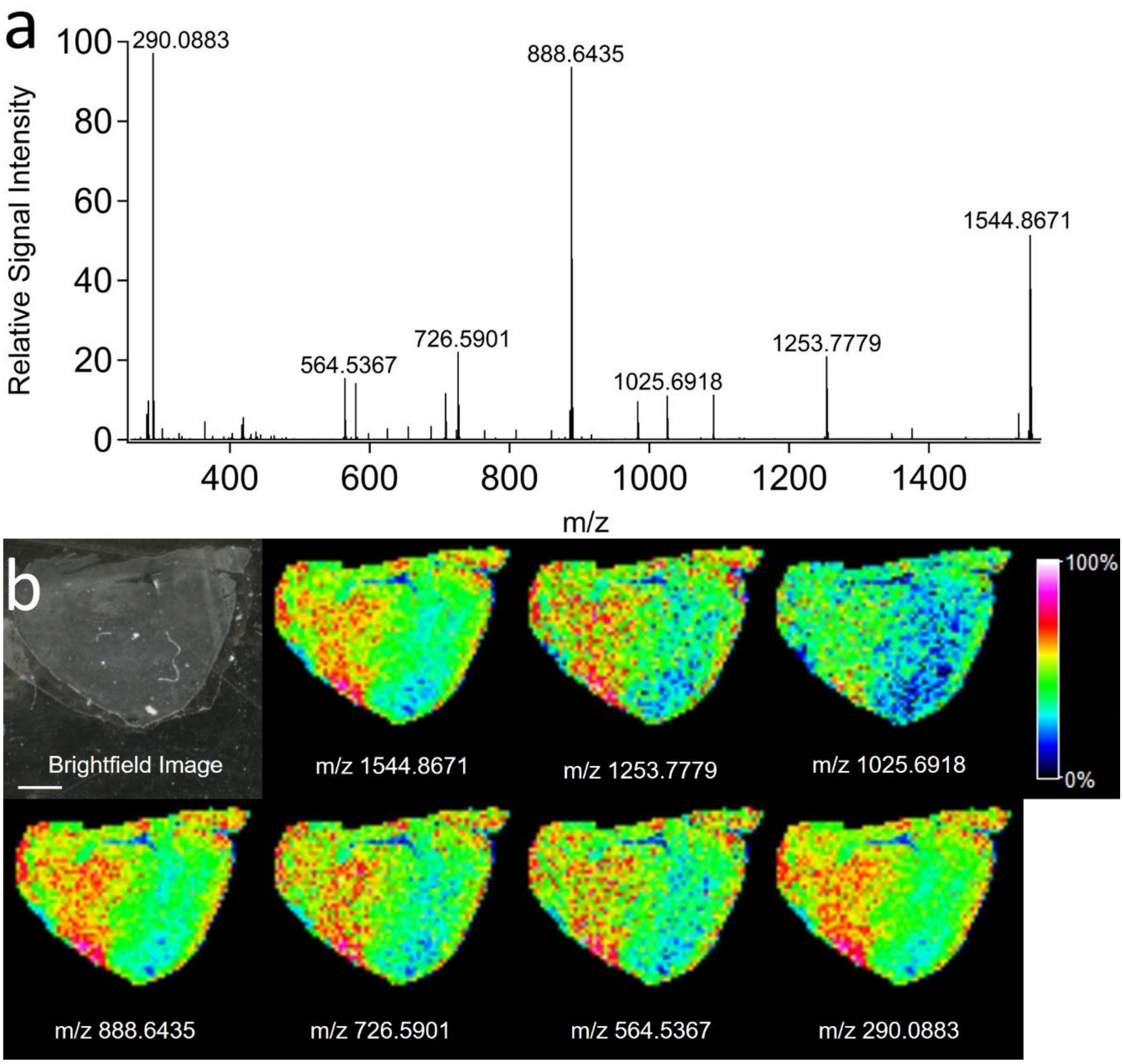

Supplementary Fig. 29. MALDI-MS/MS spectra obtained from mouse brain tissue sections using MALDI-CID-FTICR from the precursor ion at a) *m/z* 1544.8671. Fragments supporting the assignment of [GM1(36:1)-H]$^-$ are found at *m/z* 1253.7779 (loss of sialic acid from the precursor ion), *m/z* 888.6435 (loss of sialic acid, one hexose and N-Acetylgalactosamine), *m/z* 726.5961 (loss of sialic acid, two hexoses and N-Acetylgalactosamine), *m/z* 564.5367 (loss of sialic acid, three hexoses and N-Acetylgalactosamine), *m/z* 290.0883 (sialic acid). b) MALDI-MS/MS imaging of coronal mouse brain tissue section using MALDI-CID-FTICR reveals distributions of fragment ions (without normalization) at *m/z* 1253.7779 (loss of sialic acid from the precursor ion), *m/z* 888.6435 (loss of sialic acid, one hexose and N-Acetylgalactosamine), *m/z* 726.5961 (loss of sialic acid, two hexoses and N-Acetylgalactosamine), *m/z* 564.5367 (loss of sialic acid, three hexoses and N-Acetylgalactosamine), *m/z* 290.0883 (sialic acid) from the precursor ion of [GM1(36:1)-H]$^-$. Due to the isolation width of 5 *m/z*, additional isobaric and isomeric lipids are potentially also fragmented giving rise to additional product ions. Scale bar in panel (b) is 1000 µm.

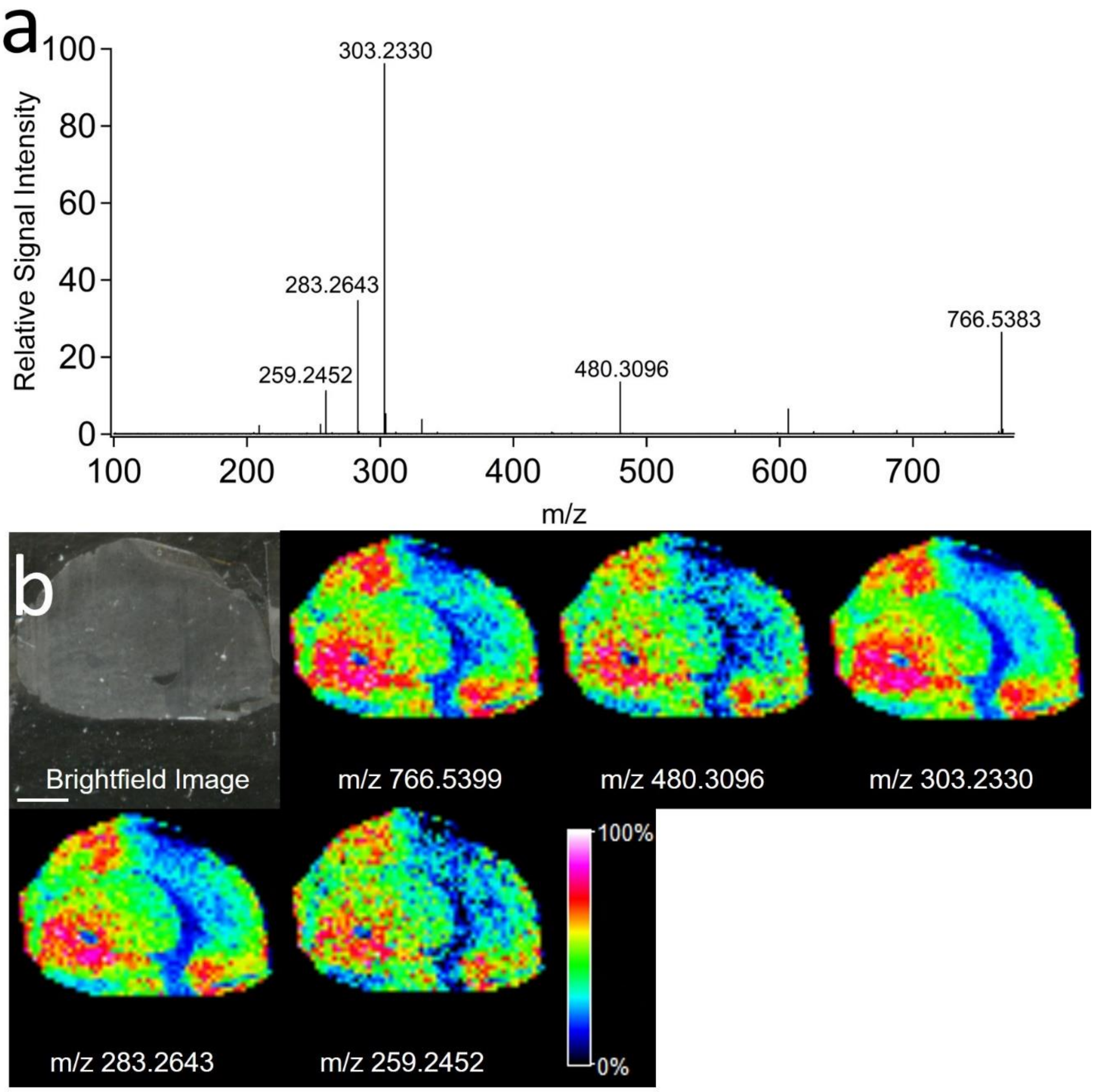

Supplementary Fig. 30. MALDI-MS/MS spectra obtained from mouse brain tissue sections using MALDI-CID-FTICR from the precursor ion at a) *m/z* 766.5383. Fragments supporting the assignment of [PE (38:4)-H]$^-$ are found at *m/z* 480.3096 (loss of sn2 acyl chain as ketene (RCH=C=O) from precursor ion), *m/z* 303.2330 (sn2 RCOO$^-$ ion), *m/z* 283.2643 (sn1 RCOO$^-$ ion), *m/z* 259.2452 (loss of CO_2 from sn2 RCOO$^-$ ion). b) MALDI-MS/MS imaging of coronal mouse brain tissue section using MALDI-CID-FTICR reveals distribution of fragment ions at *m/z* 480.3096 (loss of sn2 acyl chain as ketene (RCH=C=O) from precursor ion), *m/z* 303.2330 (sn2 RCOO$^-$ ion), *m/z* 283.2643 (sn1 RCOO$^-$ ion), *m/z* 259.2452 (loss of CO_2 form sn2 RCOO$^-$ ion). b) MALDI-MS/MS imaging of coronal mouse brain tissue section using MALDI-CID-FTICR reveals distributions of fragment ions (without normalization) at *m/z* 480.3096 (loss of sn2 acyl chain as ketene (RCH=C=O) from precursor ion), *m/z* 303.2330 (sn2 RCOO$^-$ ion), *m/z* 283.2643 (sn1 RCOO$^-$ ion), *m/z* 259.2452 (loss of CO_2 form sn2 RCOO$^-$ ion) from the precursor ion of [PE (38:4)-H]$^-$. Due to the isolation width of 1 *m/z* additional isobaric and isomeric lipids are potentially also fragmented giving rise to additional product ions. Scale bar in panel (b) is 1000 µm.

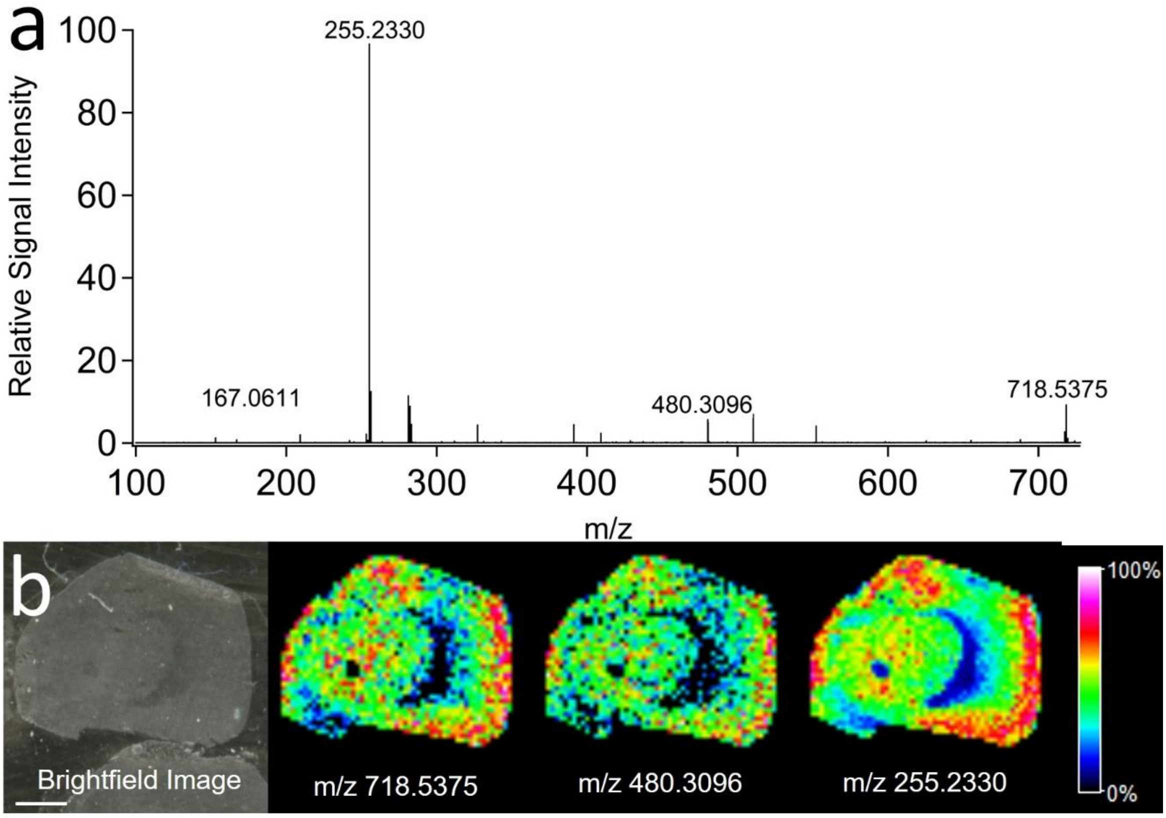

Supplementary Fig. 31. MALDI-MS/MS spectra obtained from mouse brain tissue sections using MALDI-CID-FTICR from the precursor ion at a) *m/z* 718.5375. Fragments supporting the assignment of $[PE\text{-}NMe_2(32{:}0)\text{-}H]^-$ are found at *m/z* 480.3096 (loss of sn2 acyl chain as ketene (RCH=C=O) from precursor ion), *m/z* 255.2330 (sn1 $RCOO^-$ ion), *m/z* 167.0611 (NMe_2-containing phosphatidylethanolamine head group). b) MALDI-MS/MS imaging of coronal mouse brain tissue section using MALDI-CID-FTICR reveals distributions of fragment ions (without normalization) at *m/z* 480.3096 (loss of sn2 acyl chain as ketene (RCH=C=O) from precursor ion), *m/z* 255.2330 (sn1 $RCOO^-$ ion) from the precursor ion of $[PE\text{-}NMe_2(32{:}0)\text{-}H]^-$. Due to the isolation width of 1 *m/z*, additional isobaric and isomeric lipids are potentially also fragmented giving rise to additional product ions. Scale bar in panel (b) is 1000 µm.

2. Supplementary Tables

Supplementary Table 1. Demographics and clinical information regarding donors of human substantia nigra sections

Diagnosis	Gender	Age (y)	Age of onset (y)
PD	Male	78	66
PD	Male	70	64
PD	Male	75	63
PD	Female	80	69
Ctrl	Male	73	NA
Ctrl	Male	80	NA
Ctrl	Male	77	NA
Ctrl	Female	70	NA

Y, year. Hr, hour. NA, not applicable.

Supplementary Table 2. Demographics and clinical information of PD patients and healthy controls, and PSAP and PGRN levels in CSF and plasma samples

	PD (Plasma/CSF)	PD-NC (Plasma)	PD-MCI (Plasma)	HC (Plasma/CSF)	p^a, p^b value (Plasma/CSF)
Age (y)	71.3±7.5/69.8±7.0	69.9±7.3	72.8±7.5	71.1±6.3/70.8±9.7	0.92,0.29/0.71,0.54
Gender (M&F)	32&28/14&8	16&14	16&14	10&9/8&8	0.99,0.99/0.51,0.34
Duration (y)	6.6±5.5/3.4±4.0	5.9±5.7	7.4±5.2	NA	NA
MoCA (30)	23.2±5.5/23.9±4.2	27.4±1.4	19.0±4.7	NA	NA
UPDRS (199)	53.1±19.5/46.5±16.0	45.3±15.9	60.6±19.6	NA	NA
UPDRS-Ⅲ (108)	30.5±12.3/28.0±8.6	25.4±8.2	35.4±13.7	NA	NA
MADRS-S (54)	10.6±9.2/9.4±8.6	7.3±6.3	12.1±8.7	NA	NA
HADS-A (21)	6.4±4.6/6.5±4.4	4.6±3.1	8.5±5.1	NA	NA
HADS-D (21)	4.8±4.1/5.3±4.0	3.2±2.8	6.3±4.6	NA	NA
MFS (44)	12.0±7.5/11.2±8.2	9.0±4.9	14.7±8.5	NA	NA
BDI-Ⅱ (63)	13.2±10.3/13.2±9.8	9.0±6.5	17.2±11.7	NA	NA
H&Y	2.5±1.0/2.2±0.9	2.4±0.9	2.6±1.1	NA	NA
LEDD	630.7±469.5/407.0±370.2	527.3±490.5	716.9±440.8	NA	NA
PGRN,ng/ml	33.2±6.9/2.9±0.8	32.0±7.6	34.4±6.0	30.6±3.9/3.5±0.8	*
PSAP,ng/ml	201.1±78.9/668.6±329.1	179.9±77.4	222.3±75.9	155.4±61.6/572.5±159.7	*
NfL,pg/ml	-/1526.0±256.6	-	-	-/1008.1±191.7	p = 0.17

M, male; F, female; UPDRS-III, Unified Parkinson's Disease Rating Scale-III; H&Y, Hoehn & Yahn scale; MoCA, Montreal Cognitive Assessment; MADRS-S, Montgomery-Asberg Depression Rating Scale-Self; HADS-A & HADS-D, Hospital Anxiety and Depression Scale; MFS, Mental Fatigue Scale; BDI-II, Beck Depression Inventory-II; Numbers in brackets behind each rating scale indicates the maximum score of the scale. LEDD, Levodopa equivalent daily dose; HC, healthy control; PD-MCI, PD with mild cognitive impairment; PD-NC, PD with normal cognition; p^a, PD vs. HC; p^b, PD vs. PD-MCI vs. PD-NC. Data are presented as mean ± S.E.M. Chi-square test, or Fisher's exact test was applied in gender comparisons. One-way ANOVA (three groups) or Student's t-test (two groups) was applied in age comparisons. NA: not applicable. * Refer to Supplementary Fig. 2 for group-wise comparisons.

Supplementary Table 3. Correlation matrix of PSAP and PGRN with clinical information of PD patients

Correlation statistics r/p value	PD				PD-MCI		PD-NC	
	Plasma		CSF		Plasma		Plasma	
	PSAP	PGRN	PSAP	PGRN	PSAP	PGRN	PSAP	PGRN
MoCA	-0.35/0.02	-0.04/0.76[a]	0.14/0.58	0.37/0.13	-0.17/0.40	0.34/0.09	0.41/0.07	0.07/0.78
UPDRS	0.42/0.004	-0.12/0.42	0.24/0.35	-0.35/0.16	0.47/0.02	-0.23/0.29	0.25/0.34	-0.02/0.95
UPDRS-Ⅲ	0.46/0.001	-0.03/0.82[a]	0.59/0.008[a]	-0.24/0.36	0.49/0.02	-0.27/0.20	0.26/0.29	-0.10/0.70
MADRS-S	-0.01/0.93	-0.51/0.001	0.22/0.35[a]	-0.31/0.23	-0.15/0.50	-0.73/0.0001	0.24/0.34	-0.22/0.40
BDI-Ⅱ	0.08/0.58[a]	-0.40/0.004[a]	-0.19/0.46	-0.64/0.007	-0.12/0.56[a]	-0.61/0.003	0.32/0.19	-0.43/0.03[a]
HADS-D	0.05/0.75	-0.27/0.06[a]	-0.02/0.93[a]	-0.35/0.17	-0.23/0.29	-0.51/0.01	0.16/0.54	-0.26/0.31
HADS-A	-0.19/0.24	-0.43/0.004	-0.28/0.28	-0.52/0.04	-0.50/0.02	-0.65/0.001	0.32/0.22	-0.16/0.55
MFS	0.005/0.97	-0.42/0.006	0.08/0.74[a]	-0.29/0.27	-0.25/0.26	-0.50/0.02	0.24/0.36	-0.13/0.62
H&Y	0.03/0.83	0.09/0.53	-0.19/0.46	-0.42/0.08	-0.04/0.87	0.03/0.89	0.29/0.21	0.30/0.19
LEDD	0.19/0.18	0.24/0.08	-0.05/0.84	-0.19/0.46	0.09/0.66	0.16/0.44	0.02/0.93	0.18/0.44

r, correlation coefficiency. All r/ps are Pearson's, except that r/p[a] are Spearman's.

3. Supplementary Discussion

PSAP and PGRN in biofluids

The significant correlation between PSAP and PGRN levels both in CSF and plasma confirmed the interdependence of these two proteins at individual levels. However, apart from this, these two proteins take actions independently from each other as well[3]. Consistent with this, PGRN does not fully follow the changing trajectory of PSAP in TPH^{+} neurons of cPSAP^{SERT} mice, i.e., PGRN levels do not reach WT levels while PSAP levels do in these mice, indicating a dysfunctional PSAP-independent trafficking pathway of PGRN in these neurons. Their trafficking pathways independent from each other may also contribute to their divergent correlation to Parkinson's disease (PD) symptoms. Furthermore, CSF PSAP levels do not show correlation with NfL levels, while PGRN levels do, suggestive of the presence of independent trafficking pathways or different origins of these two proteins. NfL is considered as an indicator of neuroaxonal loss and an unspecific biomarker for PD[4]. The correlation of PGRN and NfL indicates that PGRN may link to neuroaxonal loss. PGRN released by injured neurons and by glia may contribute to this correlation. However, PSAP does not link to neuroaxonal loss reflected by NfL, which means that PSAP contributes to the progression of PD in a unique way, for instance by maintaining lipid homeostasis as implicated by our study. Many cells in the nervous system release exosomes[5]. PSAP and PGRN have been shown to be released via exosomes[6, 7]. Therefore, it would be interesting to measure PSAP/PGRN in the exosomes from PD patients and explore their relationship with PD symptoms in future studies. Moreover, exosomes may provide another platform for PSAP delivery.

Lipid changes and functional outcomes

The lipid changes in desaturation levels and chain length affect a large spectrum of lipid species. As PE and PC are main phospholipid components of cellular membrane[8], CL is a signature lipid of mitochondria[9], PI is the precursor of second messengers, and sphingolipid is a critical component of membrane microdomains[10], the membrane stability/permeability/fluidity, mitochondria oxidative phosphorylation, cell signaling, and cellular events regulation of cells in brains of cPSAP^{DAT} mice may be affected consequently. The lipid dyshomeostasis is widespread in the brain of cPSAP^{DAT} mice. The

inability of SKF38393, a postsynaptic D1 agonist, to rescue LTP indicates that either pharmacological activation of postsynaptic D1 receptors is insufficient to recover LTP, or that presynaptic dopamine transmission or other neurotransmitter systems, in particular the glutamatergic/NMDA receptors[11], may be altered. A speculation is that this is an indirect effect from the strong lipid changes in the cPSAP^{DAT} mice. We present longitudinal changes in both behaviors and lipids in our study. The behavior defects are similar across different time points, while the lipid changes gradually exacerbate. This agrees with the fact that changes in markers of the dopamine system does not correlate so well with the symptoms of PD[12]. Some PD patients only show symptoms until they lose 50% of DA neurons in the SN, indicative of a non-linear relationship between pathological changes and behavioral changes[13]. Similarly, the progressive lipid changes may not necessarily linearly present as progressive behavior defects.

Total lipid classes with an emphasis on BMP

Regarding the lipid changes, limited by the technique, we could not provide absolute values for each lipid species. Often low abundant lipids show the most dramatic effect, but overall changes are neglectable. However, as shown in the CLs, high content CLs were also dramatically changes in cPSAP^{DAT} mice. Moreover, the lipid changes in desaturation and chain-length revealed in our study are two key features of all lipids; thus, most likely, common changes cover most species of lipids, including both high and low abundant ones. An analysis of total lipid classes would have provided valuable information in terms of the overall lipid changes. However, due to the limitation of detection range of our available technique, we could not calculate changes of total lipid classes. BMP is one type of negatively charged lipids and is important for the binding of lipid degradation enzymes and thus affects the metabolism of other lipids[14, 15]. Based on this feature, BMP has been proposed to be responsible for the accumulation of lipids in different studies. However, in our study, we found both increases and decreases of different lipid species, especially the decreased sphingolipids in cPSAP^{DAT} mice. Therefore, BMP may not fully explain these changes. Moreover, with MALDI-MSI experiments, we cannot separate BMP from PG. This is because BMP and PG are isomers and thus the fragments of BMP and PG are identical[15]. However, it would be interesting to study BMP in our studied mice with other

techniques, since the dramatically changed CLs share the same precursor (PG) as BMP and are extremely rich in PUFAs[15].

4. Supplementary References

1. Hsu, F.-F. & Turk, J. Structural determination of sphingomyelin by tandem mass spectrometry with electrospray ionization. *Journal of the American Society for Mass Spectrometry* **11**, 437-449 (2000).
2. Hsu, F.-F. & Turk, J. Studies on sulfatides by quadrupole ion-trap mass spectrometry with electrospray ionization: structural characterization and the fragmentation processes that include an unusual internal galactose residue loss and the classical charge-remote fragmentation. *Journal of the American Society for Mass Spectrometry* **15**, 536-546 (2004).
3. Tayebi, N., Lopez, G., Do, J. & Sidransky, E. Pro-cathepsin D, Prosaposin, and Progranulin: Lysosomal Networks in Parkinsonism. *Trends Mol Med* **26**, 913-923 (2020).
4. Parnetti, L.*, et al.* CSF and blood biomarkers for Parkinson's disease. *The Lancet. Neurology* **18**, 573-586 (2019).
5. Peng, C., Trojanowski, J.Q. & Lee, V.M. Protein transmission in neurodegenerative disease. *Nature reviews. Neurology* **16**, 199-212 (2020).
6. Toyofuku, T.*, et al.* Endosomal sorting by Semaphorin 4A in retinal pigment epithelium supports photoreceptor survival. *Genes & development* **26**, 816-829 (2012).
7. Benussi, L.*, et al.* Loss of exosomes in progranulin-associated frontotemporal dementia. *Neurobiology of aging* **40**, 41-49 (2016).
8. Yoon, J.H.*, et al.* Brain lipidomics: From functional landscape to clinical significance. *Science advances* **8**, eadc9317 (2022).
9. Ghio, S., Kamp, F., Cauchi, R., Giese, A. & Vassallo, N. Interaction of alpha-synuclein with biomembranes in Parkinson's disease--role of cardiolipin. *Prog Lipid Res* **61**, 73-82 (2016).
10. Olsen, A.S.B. & Faergeman, N.J. Sphingolipids: membrane microdomains in brain development, function and neurological diseases. *Open Biol* **7** (2017).
11. Nouhi, M., Zhang, X., Yao, N. & Chergui, K. CIQ, a positive allosteric modulator of GluN2C/D-containing N-methyl-d-aspartate receptors, rescues striatal synaptic plasticity deficit in a mouse model of Parkinson's disease. *CNS neuroscience & therapeutics* **24**, 144-153 (2018).
12. Nandhagopal, R.*, et al.* Longitudinal progression of sporadic Parkinson's disease: a multi-tracer positron emission tomography study. *Brain : a journal of neurology* **132**, 2970-2979 (2009).
13. Lang, A.E. & Lozano, A.M. Parkinson's disease. Second of two parts. *The New England journal of medicine* **339**, 1130-1143 (1998).
14. Simon, M.J., Logan, T., DeVos, S.L. & Di Paolo, G. Lysosomal functions of progranulin and implications for treatment of frontotemporal dementia. *Trends Cell Biol* (2022).
15. Fahy, E.*, et al.* Update of the LIPID MAPS comprehensive classification system for lipids. *Journal of lipid research* **50 Suppl**, S9-14 (2009).
