# Supplementary material for: Prosaposin maintains lipid homeostasis in dopamine neurons and counteracts experimental parkinsonism in rodents
Source: Nat Commun. 2023 Sep 19;14:5804. doi: 10.1038/s41467-023-41539-5 (PMC10509278; doi:10.1038/s41467-023-41539-5)
Supplement: Supplementary file 3 — Description of Additional Supplementary Files [file 41467_2023_41539_MOESM3_ESM.pdf]

Description of Additional Supplementary Files

Supplementary Data 1

Levels and statistical differences of lipids detected by mass spectrometry imaging in Caudate-Putamen (CPu), Substantia Nigra+Ventral Tegmental Area (SN+VTA), Cerebral Cortex (Ctx), Hippocampus (Hip) and Corpus Callusum (CC) from cPSAP^{DAT} KO and wildtype mice. Multiple t-tests were applied with a false discovery rate (FDR) set at 5% using the two-stage step-up method of Benjamini, Krieger, and Yekutieli.

Supplementary Data 2

Levels and statistical differences of lipids detected by mass spectrometry imaging in Cerebellum as well as comparison to changes in striatum from cPSAP^{DAT} KO and wildtype mice. Multiple t-tests were applied with a false discovery rate (FDR) set at 5% using the two-stage step-up method of Benjamini, Krieger, and Yekutieli.

Supplementary Data 3

Levels and statistical differences of lipids detected by mass spectrometry imaging in Caudate-Putamen (CPu), Dorsal Raphe Nuclei (DRN), Cerebral Cortex (Ctx), Hippocampus (Hip) and Corpus Callusum (CC) from cPSAP^{SERT} KO and wildtype mice. Multiple t-tests were applied with or without a false discovery rate (FDR) set at 5% using the two-stage step-up method of Benjamini, Krieger, and Yekutieli.

Supplementary Data 4

Levels and statistical differences of neurotransmitters detected by mass spectrometry imaging in Striatum (Str), Dorsal Raphe Nuclei (DRN), Cerebral Cortex (Ctx) and Hippocampus (Hip) from cPSAP^{SERT} KO and wildtype mice. Multiple t-tests were applied without correction.

Supplementary Data 5

Lists of statistical analyses made on experimental data presented in main and supplementary figures.

Supplementary Data 6

Data underlying Lipid Species Assignments based on ion type, chemical formula, theoretical and observed m/z ratios.
