# Supplementary material for: Prosaposin maintains lipid homeostasis in dopamine neurons and counteracts experimental parkinsonism in rodents
Source: Nat Commun. 2023 Sep 19;14:5804. doi: 10.1038/s41467-023-41539-5 (PMC10509278; doi:10.1038/s41467-023-41539-5)
Supplement: Supplementary file 10 — Reporting Summary [file 41467_2023_41539_MOESM10_ESM.pdf]

nature portfolio

Corresponding author(s): Per Svenningsson, Yachao He

Last updated by author(s): August 13, 2023

Reporting Summary

Nature Portfolio wishes to improve the reproducibility of the work that we publish. This form provides structure for consistency and transparency in reporting. For further information on Nature Portfolio policies, see our Editorial Policies and the Editorial Policy Checklist.

Statistics

For all statistical analyses, confirm that the following items are present in the figure legend, table legend, main text, or Methods section.

n/a	Confirmed	
☐	☒	The exact sample size (*n*) for each experimental group/condition, given as a discrete number and unit of measurement
☐	☒	A statement on whether measurements were taken from distinct samples or whether the same sample was measured repeatedly
☐	☒	The statistical test(s) used AND whether they are one- or two-sided<br>*Only common tests should be described solely by name; describe more complex techniques in the Methods section.*
☒	☐	A description of all covariates tested
☐	☒	A description of any assumptions or corrections, such as tests of normality and adjustment for multiple comparisons
☐	☒	A full description of the statistical parameters including central tendency (e.g. means) or other basic estimates (e.g. regression coefficient) AND variation (e.g. standard deviation) or associated estimates of uncertainty (e.g. confidence intervals)
☐	☒	For null hypothesis testing, the test statistic (e.g. F, t, r) with confidence intervals, effect sizes, degrees of freedom and P value noted<br>*Give P values as exact values whenever suitable.*
☒	☐	For Bayesian analysis, information on the choice of priors and Markov chain Monte Carlo settings
☒	☐	For hierarchical and complex designs, identification of the appropriate level for tests and full reporting of outcomes
☐	☒	Estimates of effect sizes (e.g. Cohen's *d*, Pearson's *r*), indicating how they were calculated

Our web collection on statistics for biologists contains articles on many of the points above.

Software and code

Policy information about availability of computer code

Data collection	Information of softwares used in the study is provided in the Methods section under the subheadings of different methods and techniques accordingly. Briefly, Zeiss Zen 3.1, Epson scan V750 PRO, NanoZoomer S360MD, Image J v1.53k for image collection, EthoVision XT11.5 (Noldus) for behavior tracking, Kaluza v2.1.1 for flow cytometry, Chromeleon 7 for HPLC, pClamp 10 for electrophysiology, FlexImaging v5.0 for MSI data visualization.
Data analysis	EthoVision XT11.5 (Noldus) for behavior data analysis, Image J v1.53k for image quantification, pClamp 10 for electrophysiology data analysis, SCiLS Lab v.2019a Pro for MSI data analysis, FlowJo 10.4.2 for flow cytometry data analysis; Graphpad Prism v9.3.1 and SPSS V25.0 for statistics analysis.

For manuscripts utilizing custom algorithms or software that are central to the research but not yet described in published literature, software must be made available to editors and reviewers. We strongly encourage code deposition in a community repository (e.g. GitHub). See the Nature Portfolio guidelines for submitting code & software for further information.

Data

Policy information about availability of data

All manuscripts must include a data availability statement. This statement should provide the following information, where applicable:

- Accession codes, unique identifiers, or web links for publicly available datasets
- A description of any restrictions on data availability
- For clinical datasets or third party data, please ensure that the statement adheres to our policy

All data associated with this study are presented in the paper or supplementary information. Source data underlying all figures are provided. To respect the data protection and privacy of participants, the detailed clinical information can be shared on request from qualified investigators within the limits of participants' consent and according to ethics and material transfer agreements.

Research involving human participants, their data, or biological material

Policy information about studies with human participants or human data. See also policy information about sex, gender (identity/presentation), and sexual orientation and race, ethnicity and racism.

Reporting on sex and gender	This study includes participants of both genders (male and female). Gender was determined based on self-reporting.<br>Gender information is provided in Extended Data Table 1 and 2. Chi-square test of Fisher's exact test was used for gender comparisons between/among groups.<br>Due to the relatively small sample size that has been used, gender-based analyses lack statistic power and provide little information. Therefore, no gender-based analyses have been performed.
Reporting on race, ethnicity, or other socially relevant groupings	Not applicable
Population characteristics	Detailed demographic and clinical information have been provided in Extended Data Table 1 and 2.<br>Briefly, participants of PD and control group are age and gender matched and have clear diagnosis information and medical history.
Recruitment	Formalin-fixed paraffin-embedded (FFPE) human nigra sections were obtained from the brain bank of Karolinska Institutet. Clinical ratings, cerebrospinal fluid (CSF), plasma, and peripheral blood mononuclear cells (PBMCs) were from participants enrolled in the Neurology Clinic at the Karolinska University Hospital. Family members of patients, hospital/research staff or patients without significant neurological disorders, depression, and immunological diseases were recruited as healthy controls. There was no self selection bias or other biases.
Ethics oversight	All human experiments were conducted according to the Declaration of Helsinki. Pre-mortem informed consents were signed by all donors of FFPE SNc sections, with approval from the regional ethics review board of Stockholm (2014/1366-31). Cerebrospinal fluid (CSF), plasma, and peripheral blood mononuclear cells (PBMCs) studies were approved by the regional ethics review board of Stockholm (2016/19-31/2; 2019-04967). Signed informed consents were received from all participants.

Note that full information on the approval of the study protocol must also be provided in the manuscript.

Field-specific reporting

Please select the one below that is the best fit for your research. If you are not sure, read the appropriate sections before making your selection.

[x] Life sciences [ ] Behavioural & social sciences [ ] Ecological, evolutionary & environmental sciences

For a reference copy of the document with all sections, see nature.com/documents/nr-reporting-summary-flat.pdf

Life sciences study design

All studies must disclose on these points even when the disclosure is negative.

Sample size	No sample size calculation was performed.<br>Human sample sizes have sufficient statistical power to reveal considerable positive findings as proven by the results, although it would be favorable to perform a separate larger-sample size study to further validate the results.<br>Sample sizes of animal studies were determined according to previously published studies (PMID: 25915474, 23341612) and have been proven to be adequate to measure their effect size.
Data exclusions	Due to the large heterogeneity of scores of clinical rating scales, regression outliers were detected and removed:<br>Regression outliers were define by standard residual<2σ.<br>In the correlation analysis of plasma PSAP (PD) with clinical rating scales, two patients are outliers in several non-motor rating scales (HADS-D, HADS-A, MFS, BDI-2); one patient is an outlier in HADS-A, one patient in HADS-D, and one patient in UPDRS-3; three outliers in MoCA were detected. In the correlation analysis of plasma PSAP (PD-MCI) with clinical rating scales, one outlier was detected both in HADS-A and UPDRS-3. In the correlation analysis of CSF PSAP (PD) with clinical rating scales, one outlier in UPDRS-3 was detected.

In the correlation analysis of plasma PGRN (PD) with clinical rating scales, one patient is outlier in several non-motor rating scales (HADS-D, HADS-A, MADRS-S, MFS, BDI-2), one patient is outlier in BDI-2 and MARDS-S, and these two patients are outliers in the correlation analysis of plasma PGRN (PD-MCI) with clinical rating scales. In the correlation analysis of CSF PGRN (PD) with clinical rating scales, one patient is an outlier in HADS-A.

Other outliers were detected by Grubb's test:
One outlier in Fig. 5B cKO+AAV-alpha-synuclein+AAV-PSAP group was detected and removed.
One outlier in Fig. 6E AAV-alpha-synuclein&ECB-PSAP was detected and removed.

One WT mouse in Supplementary Fig. 8D did not respond to cocaine treatment and thus were removed

Replication	All biological replicates supporting the results are indicated in each figure legend. Mice died before experiment endpoint or technically failed samples were not included. Otherwise, all replicates are successful.
Randomization	Litter mates of mice were randomly allocated into experiment groups with age and gender matched. Male rats were randomly grouped. Human samples were randomly allocated in ELISA plates.
Blinding	The investigators were blinded when collecting the data. Analysis involving manual quantification were blinded, otherwise analysis were done objectively by softwares.

Reporting for specific materials, systems and methods

We require information from authors about some types of materials, experimental systems and methods used in many studies. Here, indicate whether each material, system or method listed is relevant to your study. If you are not sure if a list item applies to your research, read the appropriate section before selecting a response.

Materials & experimental systems

n/a	Involved in the study	
☐	☒	Antibodies
☐	☒	Eukaryotic cell lines
☒	☐	Palaeontology and archaeology
☐	☒	Animals and other organisms
☒	☐	Clinical data
☒	☐	Dual use research of concern
☒	☐	Plants

Methods

n/a	Involved in the study	
☒	☐	ChIP-seq
☐	☒	Flow cytometry
☒	☐	MRI-based neuroimaging

Antibodies

Antibodies used

Information of antibodies used in the study are provided in the Methods section of the manuscript, including (dilution used, catalog no, manufacturer):
Primary antibodies:
rabbit anti-PSAP (1:100; 10801-1-AP, Proteintech)
mouse anti-PSAP (1:500; H00005660-M01, Abnova)
rabbit anti-PSAP (1:300; HPA004426, Atlas antibodies)
goat anti-PGRN (1:100; AF2420, R&D Systems)
rabbit anti-PGRN antibody, EPR18539-59 (1:50; ab187070, Abcam)
sheep anti-PGRN (1:100; AF2557, R&D systems)
mouse anti-α-synuclein (1:100; sc12767, Santa Cruz)
rabbit anti-p-Ser129 α-synuclein (1:250; ab51253, abcam)
chicken anti-GFP (1:500; ab13970, Abcam)
mouse anti-TH (1:100; 22941, Nordic Biosite)
chicken anti-TH (1:500; ab76442, Abcam)
rabbit anti-TH (1:500; AB152, Merck)
chicken anti-TH (1:500; ab76442, Abcam)
rabbit anti-TH (1:500; AB152, Merck)
rabbit anti-DAT (1:500; AB1591P, Merck)
rabbit anti-vesicular monoamine transporter 2 (VMAT2) (1:1000, 20042, ImmunoStar)
mouse anti-TPH (1:100; T0678, Sigma)
chicken anti-GFAP (1:100; ab4674, Abcam)
mouse anti-NeuN (1:100; MAB377, Chemicon)
mouse anti-Darrp32 (1:100; 611520, BD Biosciences)
goat anti-IBA1 (1:100; ab5076, Abcam)
rabbit anti-IBA1 (1:100; 019-19741, Wako)
mouse anti-Olig2 (1:100; MABN50, Merck)
rabbit anti-IDO-1 (1:100; ab106134, Abcam)

rabbit anti-TDO-2 (1:100; 15880-1-AP, Proteintech)
rabbit anti-FADS-1 (1:100; 10627-1-AP, Proteintech)
rabbit anti-SCD-1 (1:100; ab19862, Abcam)
rabbit anti-PEX14 (1:100; 10594-1-AP, Proteintech)
rabbit anti-ACOX-1 (1:100; 10957-1-AP, Proteintech)
rat anti-LAMP-1 (1:100; sc-19992, Santa Cruz Biotechnology)
goat anti-CTSD (1:100; AF1029, R&D systems)
rabbit anti-GBA (1:100; ab128879, Abcam)
rabbit anti-UGCG-1 (1:100, 12869-1-AP, Proteintech)
rabbit anti-GALC (1:100, 11991-1-AP, Proteintech)
rabbit anti-GLB-1 (1:100, 15518-1-AP, Proteintech)
rabbit anti-HEX-A (1:100; 11317-1-AP, Proteintech)
rabbit anti-SGMS-1 (1:100; 19050-1-AP, Proteintech)
rabbit anti-SMPD-1 (1:100; 14609-1-AP, Proteintech)

Fluorescent secondary antibodies:
donkey anti-mouse IgG Alexa Fluor 488 (1:500; A21202, Invitrogen)
donkey anti-sheep IgG Alexa Fluor 488 (1:500; ab150177, Abcam)
donkey anti-goat IgG Alexa Fluor 568 (1:500; A11057, Invitrogen)
donkey anti-rabbit IgG Alexa Fluor 568 (1:500; A10042, Thermo Fisher Scientific)
donkey anti-rabbit IgG Alexa Fluor 647 (1:500; 711-605-152, Jackson Immuno-Research)
donkey anti-mouse IgG Alexa Fluor 647 (1:500; A32787, Thermo Fisher Scientific)
CD3 FITC clone UCHT1 (1:50; 11-0038-42, Thermo Fisher Scientific)
CD16 PerCP clone 3G8 (1:30; MHCD1631, Thermo Fisher Scientific)
CD14 BV510 clone M5E2 (1:30; 348807, Biolegend)
CD4 FITC clone OKT4 (1:50; A27064, Thermo Fisher Scientific)
CD3 PerCP/Cy5.5 clone UCHT1 (1:30; 300430, Biolegend)
CD8 BV510 clone SK1 (1:30; 563919, BD biosciences)
APC-conjugated goat anti-rabbit antibody (1:100; F0111, R&D systems)

Biotinylated secondary antibodies:
goat anti-rabbit IgG (1:300, BA1000, Vector Laboratories),
goat anti-chicken IgG (1:300; SAB3700204, Merck)
horse anti-mouse IgG (1:300; BA2001, Vector Laboratories)

Validation

Validation information and images of antibodies are available on the manufacturer's website and citations, as listed:
rabbit anti-PSAP (human, mouse) (10801-1-AP, Proteintech), https://www.ptglab.com/products/PSAP-Antibody-10801-1-AP.htm, PMID:27356620
mouse anti-PSAP (human)(H00005660-M01, Abnova), https://www.abnova.com/products/products_detail.asp?Catalog_id=H00005660-M01, PMID: 19393779
rabbit anti-PSAP (human)(HPA004426, Atlas antibodies), https://www.atlasantibodies.com/products/antibodies/primary-antibodies/triple-a-polyclonals/psap-antibody-hpa004426/, PMID: 31600775
goat anti-PGRN (human)(AF2420, R&D Systems),https://www.rndsystems.com/products/human-progranulin-pgrn-antibody_af2420, PMID: 31864418
rabbit anti-PGRN (mouse)(ab187070, Abcam), https://www.abcam.com/granulin-antibody-epr18539-59-ab187070.html, PMID: 33390170
sheep anti-PGRN (mouse)(AF2557, R&D systems), https://www.rndsystems.com/products/mouse-progranulin-pgrn-antibody_af2557, PMID: 35169707
mouse anti-α-synuclein (human)(sc12767, Santa Cruz), https://www.scbt.com/p/alpha-synuclein-antibody-211, PMID: 33753734
rabbit anti-p-Ser129 α-synuclein (human)(ab51253, abcam), https://www.abcam.com/products/primary-antibodies/alpha-synuclein-phospho-s129-antibody-ep1536y-ab51253.html, PMID: 37193692
chicken anti-GFP (ab13970, Abcam), https://www.abcam.com/gfp-antibody-ab13970.html, PMID: 35007757
mouse anti-TH (human, mouse, rat)(22941, Nordic Biosite), https://www.immunostar.com/product/tyrosine-hydroxylase-antibody/#, PMID: 35301315
chicken anti-TH (mouse)(ab76442, Abcam), https://www.abcam.com/tyrosine-hydroxylase-antibody-ab76442.html, PMID: 35277506
rabbit anti-TH (human, mouse, rat)(AB152, Merck), https://www.sigmaaldrich.com/SE/en/product/mm/ab152, PMID: 35219402
rabbit anti-DAT (mouse, rat)(AB1591P, Merck), https://www.sigmaaldrich.com/SE/en/product/mm/ab1591p, PMID: 32955434
rabbit anti-vesicular monoamine transporter 2 (VMAT2) (rat)(20042, ImmunoStar),https://www.immunostar.com/product/vesicular-monoamine-transporter-2-rabbit-antibody/#, PMID: 29184069
mouse anti-TPH (mouse) (T0678, Sigma), https://www.sigmaaldrich.com/SE/en/product/sigma/t0678, PMID: 33247179
chicken anti-GFAP (mouse, rat)(ab4674, Abcam), https://www.abcam.com/gfap-antibody-ab4674.html, PMID: 35437315
mouse anti-NeuN (human, mouse, rat) (MAB377, Chemicon), https://www.sigmaaldrich.com/SE/en/product/mm/mab377, PMID: 35534531
mouse anti-Darrp32 (mouse, rat)(611520, BD Biosciences), https://www.bdbiosciences.com/en-us/products/reagents/microscopy-imaging-reagents/immunofluorescence-reagents/purified-mouse-anti-darpp-32.611520, PMID: 34348999
goat anti-IBA1 (ab5076, Abcam), https://www.abcam.com/iba1-antibody-ab5076.html, PMID: 35426376
rabbit anti-IBA1 (human, mouse, rat)(019-19741, Wako), https://labchem-wako.fujifilm.com/us/product/detail/W01W0101-1974.html, PMID: 20404134
mouse anti-Olig2 (human, mouse, rat)(MABN50, Merck), https://www.sigmaaldrich.com/SE/en/product/mm/mabn50, PMID:

34549820
rabbit anti-IDO-1 (human, mouse, rat)(ab106134, Abcam), https://www.citeab.com/antibodies/767184-ab106134-anti-indoleamine-2-3-dioxygenase-antibody?des=00c7ba6664eb2467, PMID: 32187541
rabbit anti-TDO-2 (human, mouse, rat)(15880-1-AP, Proteintech), https://www.ptglab.com/products/TDO2-Antibody-15880-1-AP.htm, PMID: 33951424
rabbit anti-FADS-1 (human, mouse)(10627-1-AP, Proteintech), https://www.ptglab.com/products/FADS1-Antibody-10627-1-AP.htm, PMID: 34986331
rabbit anti-SCD-1 (human)(ab19862, Abcam), https://www.abcam.com/products/primary-antibodies/scd1-antibody-cde10-ab19862.html, PMID: 34987154
rabbit anti-PEX14 (human, mouse, rat)(10594-1-AP, Proteintech), https://www.ptglab.com/products/PEX14-Antibody-10594-1-AP.htm, PMID: 33152269
rabbit anti-ACOX-1 (human, mouse, rat)(10957-1-AP, Proteintech), https://www.ptglab.com/products/AOX-Antibody-10957-1-AP.htm, PMID: 36629048
rat anti-LAMP-1 (mouse)(sc-19992, Santa Cruz Biotechnology), https://www.scbt.com/p/lamp-1-antibody-1d4b, PMID: 36202848
goat anti-CTSD (mouse)(AF1029, R&D systems), https://www.rndsystems.com/products/mouse-cathepsin-d-antibody_af1029?utm_source=citeab&utm_medium=referral&utm_campaign=product&utm_term=primaryantibodies, PMID: 35977928
rabbit anti-GBA (rat, human)(ab128879, Abcam), https://www.abcam.com/products/primary-antibodies/gba-antibody-epr51433-ab128879.html, PMID: 37045813
rabbit anti-UGCG-1 (human, mouse)(12869-1-AP, Proteintech), https://www.ptglab.com/products/UGCG-Antibody-12869-1-AP.htm, PMID: 36709325
rabbit anti-GALC (human, mouse, rat)(11991-1-AP, Proteintech), https://www.ptglab.com/products/Galc-Antibody-11991-1-AP.htm, PMID: 32501606
rabbit anti-GLB-1 (human, mouse)(15518-1-AP, Proteintech), https://www.ptglab.com/products/GLB1-Antibody-15518-1-AP.htm, PMID: 36144658
rabbit anti-HEX-A (human, mouse)(11317-1-AP, Proteintech), https://www.ptglab.com/products/HEXA-Antibody-11317-1-AP.htm, PMID: 19144319
rabbit anti-SGMS-1 (human, mouse, rat)(19050-1-AP, Proteintech), https://www.ptglab.com/products/SGMS1-Antibody-19050-1-AP.htm, PMID: 35230716
rabbit anti-SMPD-1 (human, mouse, rat)(14609-1-AP, Proteintech), https://www.ptglab.com/products/SMPD1,ASM-Antibody-14609-1-AP.htm, PMID: 35391788

Eukaryotic cell lines

Policy information about cell lines and Sex and Gender in Research

Cell line source(s)	ARPE-19, ATCC; N2a cells were a gift from Dr Patrick Allen, Rockefeller University
Authentication	Not authenticated in this study
Mycoplasma contamination	Mycoplasma contamination is tested and negative.
Commonly misidentified lines (See ICLAC register)	Not used in this study

Animals and other research organisms

Policy information about studies involving animals; ARRIVE guidelines recommended for reporting animal research, and Sex and Gender in Research

Laboratory animals	Mice: C57BL/6J, age (2m, 4m, 8m, 16m) and sex matched (male and female). Rats: Sprague Dawley, 10wks, male.
Wild animals	No wild animals were used in the study.
Reporting on sex	This study apply to both sexes, since male and females were included in the experiments. Chi-square test was used to make sure sex is matched in different groups. Due to the relatively small sample size that has been used, sex-based analyses lack statistic power and provide little information. Therefore, no sex-based analyses have been performed.
Field-collected samples	No field collected samples were used in the study.
Ethics oversight	Studies on mice were approved by local ethical committee at Karolinska Institute (1525-2017) and conducted in accordance with the European Communities Council Directive of 24 November 1986 (86/609/EEC). Studies on rats were approved by local Animal Ethics Committee at Karolinska Institutet (5018-2018).

Note that full information on the approval of the study protocol must also be provided in the manuscript.

Flow Cytometry

Plots

Confirm that:

- [x] The axis labels state the marker and fluorochrome used (e.g. CD4-FITC).
- [x] The axis scales are clearly visible. Include numbers along axes only for bottom left plot of group (a 'group' is an analysis of identical markers).
- [x] All plots are contour plots with outliers or pseudocolor plots.
- [x] A numerical value for number of cells or percentage (with statistics) is provided.

Methodology

Sample preparation	Sample preparation details have been given in the Methods section. Briefly, frozen PBMCs were quickly thawed and washed with PBS. Viability staining was performed with a near-IR dead cell marker (Invitrogen, L10119), followed by washing and incubation in blocking buffer (1% mouse serum and 1% FBS in PBS). Cells were incubated in a mixture of antibodies to identify different cell types. Upon washing, cells were fixed and permeabilized with Cytofix/Cytoperm solution (BD, #554714) for 20 min, followed by subsequent primary antibody and secondary antibody staining.
Instrument	Gallios (Beckman Coulter)
Software	Kaluza v2.1.1 and FlowJo v10.4.2
Cell population abundance	At least 10,000 cells/sample were counted and analyzed.
Gating strategy	Gating strategies for T cell and monocytes are provided in the Extended Data Fig. 3.

- [x] Tick this box to confirm that a figure exemplifying the gating strategy is provided in the Supplementary Information.
